# School-based social and behavior change communication (SBCC) advances community exposure to malaria messages, acceptance, and preventive practices in Ethiopia: A pre-posttest study

**DOI:** 10.1371/journal.pone.0235189

**Published:** 2020-06-25

**Authors:** Yohannes Kebede, Lakew Abebe, Guda Alemayehu, Morankar Sudhakar, Zewdie Birhanu

**Affiliations:** 1 Department of Health, Behavior, and Society, Jimma University, Jimma, Ethiopia; 2 President’s Malaria Initiative, United States Agency for International Development, Addis Ababa, Ethiopia; Faculty of Science, Ain Shams University (ASU), EGYPT

## Abstract

**Background:**

Ethiopia has shown incredible success in malaria morbidity, mortality, and control. Community empowerment is a milestone to meet the ambitious plans of eliminating malaria by 2030.

**Objectives:**

This study aimed to evaluate school-based malaria social behavior change communication (SBCC) in terms of community message exposure, acceptance, knowledge, and practices.

**Methods:**

A community-based pre-posttest study was conducted in five districts of the Jimma Zone, Ethiopia. 762 and 759 households were sampled for baseline and end-line, respectively. The intervention engaged students from primary schools on participatory peer education within small groups, followed by exposing parents with malaria messages aimed to influencing ideation and preventive practices. The data were analyzed using Statistical Package for Social Sciences (SPSS) version 20.0. Proportion/mean differences were computed to compare both surveys on exposure, knowledge, perceptions, and practices at p <0.05. Finally, a regression analysis was conducted between key changes and school-based exposure.

**Results:**

The study revealed a sharp increase in exposure to malaria messages with effect size (ES) of 65.7%, p <0.001. School specific exposure has grown to 57.8% (ES = 44.5%). Comprehensive knowledge about malaria increased to 39.1% (ES = 14.8%). Identifying mosquito bites as a cause of malaria was increased by ES = 20.8%. A slight reduction in risk perception (ES = 3.3%) and attitude (ES = 3.8%) and a higher rise in self-efficacy (ES = 8.5%) were observed. Community message acceptance in favor of malaria danger control was significantly improved by 10% i.e. an increase in rational decision making to uptake preventive practices. Consistently, insecticide-treated nets (ITNs) usage was improved to 63.0% (ES = 25.8%). Giving priority to use ITNs for under five years old children and pregnant women grew by 16.3% and 24.8%, respectively. Significant improvements were observed in treatment-seeking for fever (ES = 16.3%) and early treatment-seeking (ES = 15.5%). Not painting or plastering walls 6 months within spraying changed by ES = 61%. No significant change was observed in drug adherence. The school-based content intensity of exposure had effects on comprehensive knowledge, message acceptance, and ITN utilization.

**Conclusions:**

Engaging school-aged children effectively advances community exposure, perception, and behaviors. We recommend the inclusion of school-based SBCC in the national malaria control programs.

## Introduction

Globally, malaria transmission has shown a significant reduction [1]. However, it remains a public health challenge. For example, nineteen countries in sub-Saharan Africa and India carried almost 85% of the global malaria burden [2, 3]. Currently, the global malaria program aims to achieve a country by country elimination by 2035 and global eradication (2040–2050) [4–6]. Ethiopia has been implementing national malaria strategic plans (NMSPs) phase III aiming to meet the ambitious goal of eliminating malaria in 50 districts by 2020 and entirely by 2030 [7–12]. The Ethiopian National Malaria Indicator Survey (ENMIS) 2015 revealed that 65% of districts in the country were malarious, and 53% had a risk of moderate to high transmission [13–16]. The road map for global elimination plans recommends the integration of policies, resources, frameworks, and diversified strategies that are context-specific, theory, and evidence-supported to intensify interventions, particularly in high transmission settings [7–9].

The global malaria control strategy has shown progressive commitments in providing services and resources required to enhance the move towards malaria elimination [2–5]. For example, insecticide-treated nets (ITNs), indoor residual spray (IRS), accurate diagnosis, prompt treatment by artemisinin-based combination therapies (ACTs), and intermittent preventive treatment of pregnant women (IPTp) are some of the effective services [1–6]. Accordingly, in Ethiopia, across the NMSP phases (I—III), key malaria prevention and control services have intensified: ACTs, expansion of the use of rapid diagnostic tests (RDT) by health extension workers (HEWs) in health posts, vector control, and prevention through the wide distribution of ITNs and IRS. Moreover, to maximize the benefits of service support, the leading role of the NMSP has adapted key intervention strategies: community empowerment and mobilization, diagnosis of all suspected cases, management of confirmed malaria cases, prevention/vector control, surveillance and response, and monitoring and evaluation [13–18].

The Roll Back Malaria (RBM) and the World Health Organization (WHO) malaria elimination framework have devised several behavioral indicators. The social and behavior change communication (SBCC) indicators aim to enhance community empowerment and mobilization towards malaria prevention, control, and uptake of the services [12, 15, 17]. The community’s malaria preventive behavior indicators were aligned with the ITNs and IRS (core mosquito vector control methods), habitat modification (supplementary control strategy), and seeking treatment within 24 hours of the onset of fever, and adherence to anti-malarial drugs given by health workers (case management) [19–22]. Therefore, consistent with the RBM-SBCC indicators, ENMSP III delineated: community exposure to messages, knowledge, risk appraisals, perception of response efficacies regarding the use of ITNs, environmental manipulation, and early treatment-seeking for fever for a move towards malaria elimination from Ethiopia by 2030 [7–17].

Currently, the world is experiencing a shift towards making the SBCC part of any effective programs. SBCC is an approach that is evidence-based and theoretically supported and makes use of any opportunity for communication. For example, it utilizes locally available communication resources like mass media (radio, TV, etc.), m-health (mobile platforms), organizations (schools, health, religious, etc.), inter-personal communications (health workers-community members, teachers-students, student-parents, peer discussions, etc.), and community-based channels (community gatherings, meetings, social networks, traditional means like poems, proverbs, etc.) [22–26]. Evidence reports SBCC as a relevant tool for averting and controlling many forms of public health challenges [19–22, 35].

It has also been one of the evident approaches for a move towards the achievement of malaria prevention, control, and elimination through shifts in norms, knowledge, and practices. For example, various forms of interventions across different contexts reported changes in behaviors: utilization of ITN and treatment-seeking were the major ones [26–35]. Studies have reported that the effectiveness of SBCC is significant when communication is supported by access to resources (ITNs, IRS, and treatment facilities and tools) required to execute the target malaria practices [33, 36–41]. Moreover, the involvement of organizations (schools, religious centers, community meetings, social networks) has been effective for disease prevention and health promotion [22–25]. Through schools, many countries have produced economic health improvements via students and the community’s exposure to behavior change messages [26–32].

Globally, school health initiatives have encompassed strategies aimed to improving the capacity, knowledge, and decision-making skills that help to promote health and prevent diseases among school children, their families, and beyond [42–45]. In this regard, school students were also perceived to play a pivotal role in keeping the health of their families and communities. Malaria is one of the focal topics of the school health program globally [23–25, 42–45]. Currently, RBM monitoring and evaluation programs seek to intensify various SBCC strategies by assessing their outcomes [18, 19]. This study hypothesized that school-based SBCC is an effective strategy for malaria prevention and control. It was focused on enhancing the community’s malaria prevention and control skills by empowering primary schools through trained teachers, facilitating students' peer education, and ultimately reaching out to their families and neighbors with messages. Therefore, this study aimed to assess the change in households’ malaria message exposure, acceptance, and behaviors through empowered primary school students.

## Methods and materials

### Study settings

**Fig 1:**
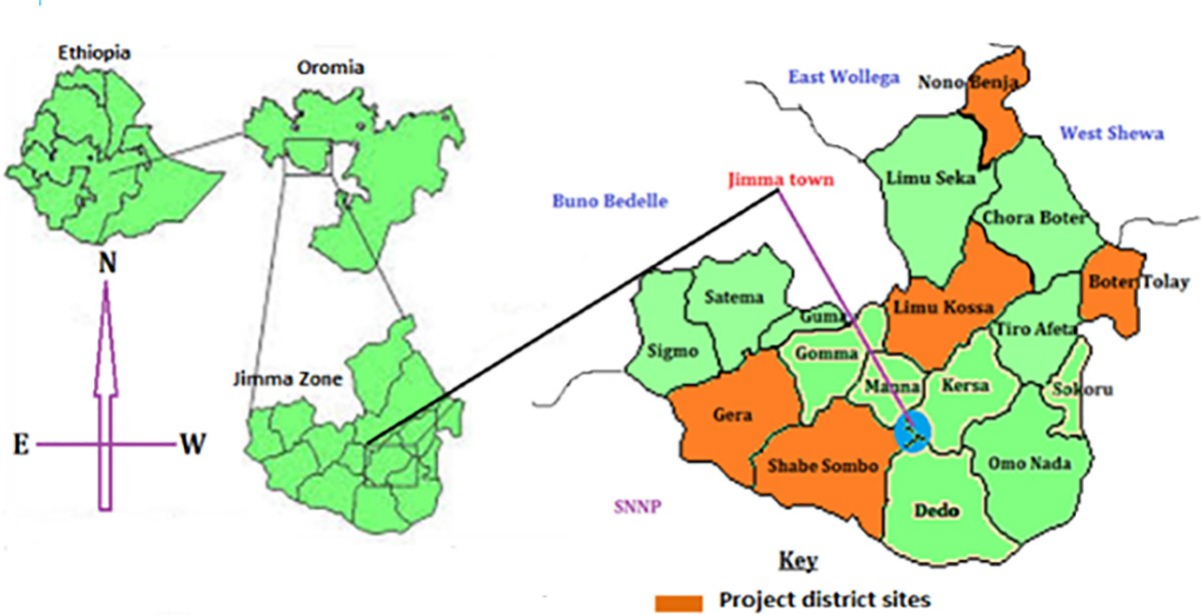
Map of the Jimma zone and target districts in Oromia, Ethiopia (Adapted from Google, Wikipedia).

The data were collected in two phases (baseline, 2017) and (end-line, 2019) in five districts of Jimma zone namely; Limmu-Kossa, Botor-Tolay, Gera, Shebe-Sombo, and Nono-Benja. Fig 1 refers to the map of the study area. The districts were selected for the SBCC based on the malaria annual parasite incidence (API) of the Jimma zone in 2016. In consultation with health offices, we further selected 75 gandas (administrative units that make up a district): 15 for each district. The 75 gandas corresponded to 75 schools involved in the project. Therefore, most of the selected villages in the districts were labeled as high-medium-malaria-burden strata. The districts are at 70–229 K.M distance from Jimma town, the capital of the zone.

### Study design and period

The study used a pre-posttest design with cross-sectional household surveys. The baseline survey was conducted in October—2017 and the end line in March—2019. The same settings and procedures were used for both surveys. Once the baseline survey was completed, we launched the school-based SBCC intervention activities in January 2018 that continued until May 2019. Throughout the intervention period, community members received packages of malaria education through trained school students.

### Sample size

Community-based surveys were conducted among heads of households. If the head of the household was unavailable, spouses were interviewed. Sample sizes for the two surveys were determined using two population proportion formula [(n = (Z_α/2_+Z_β_)^2^ * (P_1_(1-P_1_)+P_2_(1-P_2_)) / (P_1_-P_2_)^2^], where Z_α/2_ is the critical value of the normal distribution at α/2, at a confidence level of 95%, which gives a critical value of 1.96), Z_β_ is the critical value of the normal distribution at β (for a power of 80%, β is 0.2, and the critical value is 0.84), and to detect a minimum difference (P_1_-P_2_) of 7.5% between the two proportions (baseline and end-line) in ITN usage, where P_1 =_ 38.0% was taken from a previous study in similar districts. ITN usage was chosen because it is one of the key indicators in malaria prevention, the core vector control method to be promoted by RBM and national malaria elimination plans. Accordingly, the calculation yielded a total sample of 762 households for both surveys. 762 HHs responded to the baseline survey, while 759 to the end-line. The HHs surveyed at an end-line were different from the baseline ones.

### Sampling technique

We used a multi-stage sampling method. First, based on the probability proportional to the size of the total population, the sample size was allocated to each study district and ganda. Considering the logistic issue, 20 gandas were randomly selected [4 from each district). In each ganda, three subdivisions (zoni) were represented in the study based on probability proportional to the sizes of total households in the selected gandas. Accordingly, the following HHs samples were allocated to the districts: Botor-Tolay, 120; Nono-Benja, 124; Limmu-Kossa, 132; Gera, 167; and Shebe-Sombo, 219. Every ganda has three zoni. The households in zoni were counted from registration books located in the respective gandas. Finally, the households were selected using computer-generated random numbers produced based on the number of households in each zoni. Finally, the homes of the selected households were traced through local guides.

### Intervention packages and process

**Fig 2:**
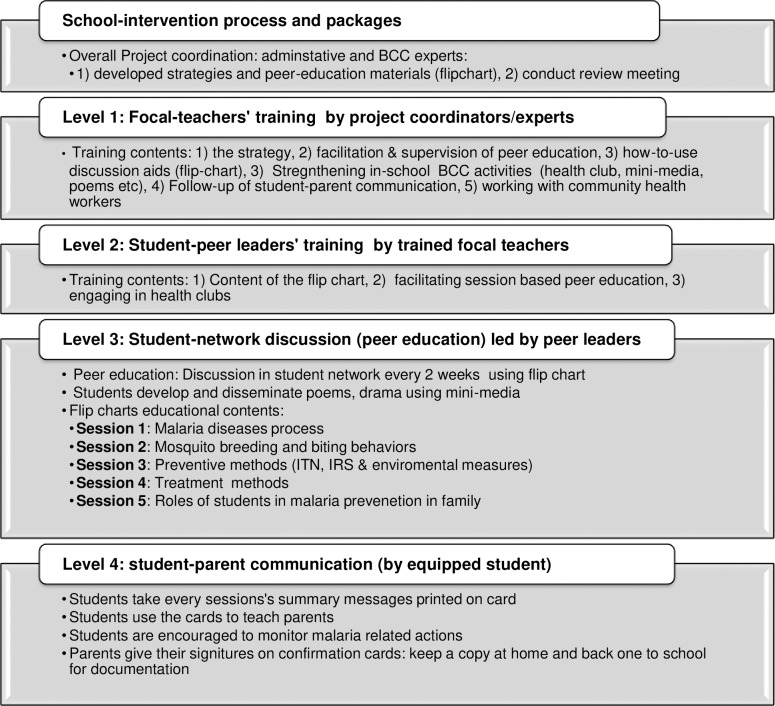
Presents the intervention processes and package, school based SBCC, Jimma, Ethiopia, 2017–19.

The SBCC strategy being evaluated was aimed at investigating changes in household’s exposure to and recall malaria messages, perceptions, and practices through organized intervention in the school community. It consisted of engaging students in primary schools (5–8 grades) located in target settings to reach out to their respective families. The current Ethiopian education system considers the peer learning approach, where every student in primary schools was supposed to be a member of a student circle (also called student army, i.e., 1 to 5 networks) [46]. Fig 2 provides a graphical presentation of the intervention package and process. Each student circle in every classroom has a group (army) leader responsible for facilitating peer education under close supervision by focal teachers. The army leaders were academically top performers. In these schools, there were two forms of facilitators’ training. First, selected natural science teachers were trained to be school-level malaria SBCC focal persons (facilitators). Second, the student army leaders received training of trainees by these facilitators. The content of training included the basics of malaria such as cause, manifestations, prevention measures, and roles of the students. Moreover, essential malaria messages and do-able actions were given due attention during training. Besides, anti-malaria club members have undergone similar pieces of training. After the training, the army leaders conducted peer-to-peer education among their group members. The peer education guiding flipchart has five separate session topics to complete the package. Ultimately, the students educating their parents were jointly followed by focal teachers and the project's local coordinators. There was a confirmation card that parents should give their signature on, send back to school for documentation, and cross-checking after the students have taught them the messages from every peer discussion session.

## Conceptual framework for malaria SBCC towards community behavior changes

**Fig 3:**
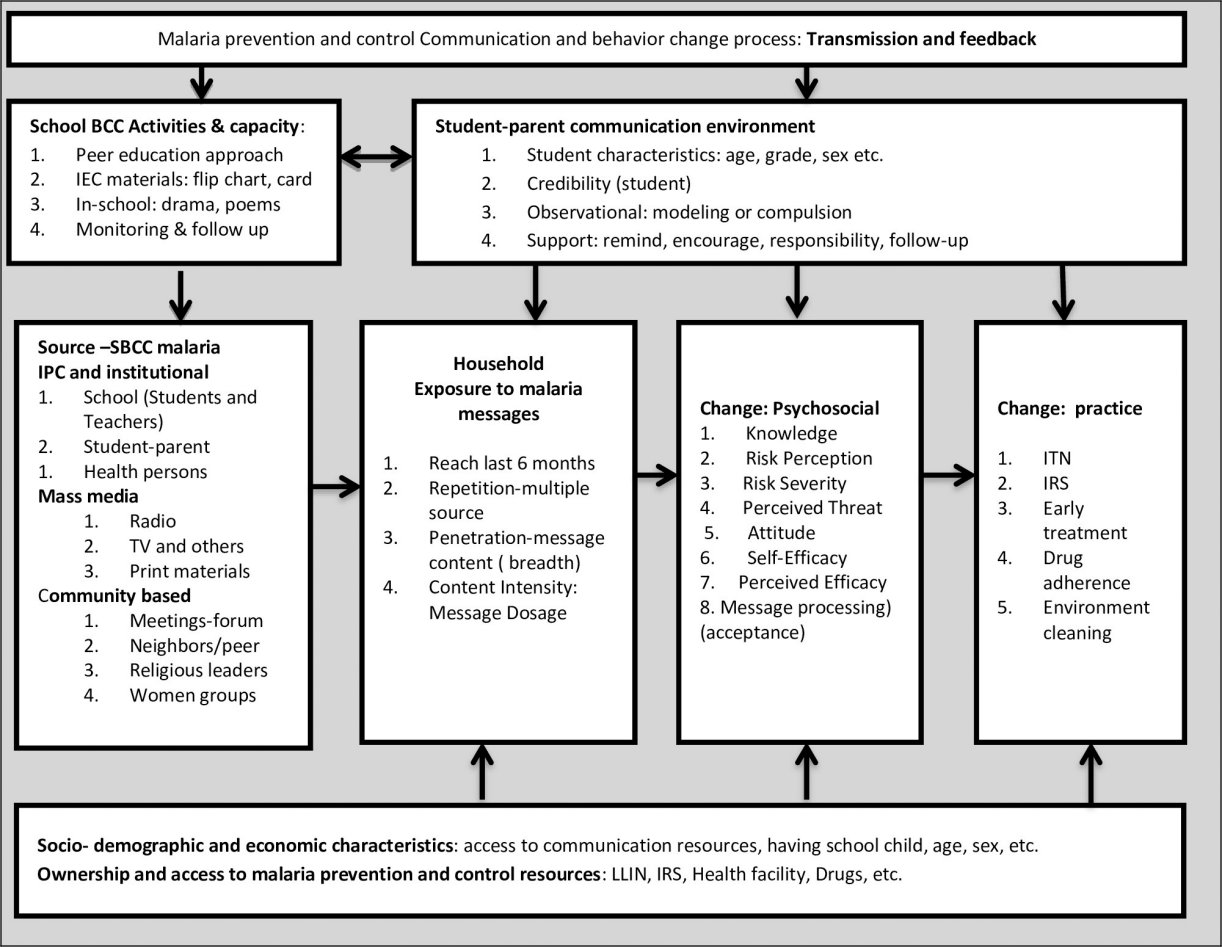
School-based malaria SBCC framework.

Fig 3 presents a conceptual framework for school-based malaria SBCC aimed at producing changes in households’ malaria message exposure, acceptance, knowledge, and preventive actions.

### Measurements

#### Outcome variables

In this study, changes in the community’s exposure to malaria messages through schools were expected to produce changes in knowledge, message processing and perceptions (susceptibility, severity, attitude, self-efficacy), message acceptance (perceived efficacy minus perceived threat = message acceptance in favor of danger versus fear control), and five key preventive practices (sleeping under ITN every night, healthy IRS handling, environmental cleaning, seeking treatment from health facilities within 24 hours of the onset of fever, and completing the anti-malaria drugs provided by health professionals). These variables were also the RBM's SBCC indicators. Resources (ITNs, IRS, and anti-malarial drugs) required to enable execution of the practices were provided through the health systems: health posts that are located in every ganda (village) and coordinated through malaria prevention and control officers at the district health office. The task of this SBCC was to enhance community consciousness and concern while improving integration between schools and health sectors towards advanced community malaria preventive practices. Therefore, the SBCC project was operating to advance malaria preventive practices, perceptions, and knowledge.

#### Instrument

Standard tools were adopted from RBM SBCC indicators and other similar studies [3–5, 18–21]. Necessary revisions were done to the tools after pretesting. A similar questionnaire was used for both surveys. Some variables were measured only at the end-line. The questionnaires consisted of several parts. Part I: socio-demographic characteristics (age, sex, education, occupation, marital status, family size, etc.). Part II: respondents’ knowledge about malaria and its prevention and control practices measured through ‘yes’ or ‘no’ formats. Part III: attitude and risk perceptions (attitude: 16 items [8 behavioral beliefs and 8 values], self-efficacy: 7 items, perceived susceptibility: 7 items, and perceived severity: 5 items) measured using a three-point Likert scale. Part IV: respondents and household members’ malaria prevention practices: ITN utilization, treatment-seeking for fever within 24 hours of onset, IRS and handling practice, compound and surrounding habitat manipulations, and drug adherence.

#### Data collectors and supervisors

The data were collected through face-to-face interviewer-administered methods at the household level. A total of 12 experienced enumerators (6 each males and females) participated in collecting the data. They were diploma to bachelor’s degree holders and fluent in Afaan Oromo as a local language. Three days of training were given to enumerators and supervisors about the purpose of the study, instruments, and data collection procedures during both surveys. The data collection process was closely supervised by the research teams. The questionnaires were checked daily for completeness and consistency. The interviewers and respective supervisors addressed the questions of overseeing investigators in their daily audits.

### Operational definitions

#### Exposure

A multi-dimensional recall of malaria messages: reach recalls about any exposure in the last 6 months; repetition, recalls about all sources of exposure over the last 6 months, and intensity (dosage) recalls about penetration by specific contents or the summations of contents over the last 6 months. Repetition is the proportion of person-to-all message sources count to which a respondent was exposed over the last 6 months. Content intensity (dosage) is the proportion of person-to-all messages count to which a respondent was exposed over the last 6 months. The above definition yields weighted scores of repetition and content intensity.

#### Comprehensive knowledge

Summed-up from correct answers given to the nine most important knowledge items about malaria signs (3), cause (1), prevention measures (3), and high-risk groups (2). Highly knowledgeable refers to the proportion of respondents giving the 9 correct answers from exhaustive counts of those elements.

#### Attitude (response efficacy)

The multiplicative weight of behavioral beliefs on key malaria practices with their respective values. In this case, we scored an attitude from every 8 distinct beliefs and values, each. Every item evaluated respondents on a three-point Likert scale. Accordingly, the score ranged from 8 to 72, with a higher score implies a favorable attitude toward malaria prevention actions. Then, to make a comparison of effect sizes with other psychological constructs possible, we weighted the score by multiplying it by 100, divided by the maximum score. The weighted mean score refers to the respondents with a favorable attitude. Cronbach’s alpha score for the belief items was 0.87, indicating good reliability for belonging to attitude as a construct.

#### Perceived threat

First, we built perceived susceptibility and severity from corresponding reliable items. The Cronbach’s alpha scores were 0.79 and 0.84 for perceived susceptibility and severity. Then, all valid items of perceived susceptibility and severity were summed, multiplied by 100, and divided by a maximum summation score was finally yielding a weighted score of the perceived threat from malaria.

#### Perceived efficacy

Weighted scores of attitude (response efficacy) and self-efficacy were summed up and divided by two to create a weighted score of perceived efficacy of malaria preventive actions.

#### Message acceptance

Overall messages to which respondents were exposed initiated psychological processing with two alternatives of message acceptance. Alternative 1: excess perceived threat compared to perceived efficacy messages that labeled respondents in the fear control zone. Alternative 2: excess perceived efficacy compared to perceived threat messages that labeled respondents in the danger control zone. According to the extended parallel process model of communication [47], people in fear control become reluctant to engage in malaria preventive actions even in the presence of resources (ITN, IRS, and treatments), while people in danger control strive utmost towards the uptake of preventive actions.

#### Discriminative value (DV)

A value used to discriminate fear from danger control. Positive and negative DV indicate danger and fear control, respectively. DV was computed as perceived efficacy minus perceived threat.

#### Data management and analysis

The data were analyzed using SPSS version 20.0. Before the analysis, the data were cleaned and checked for completeness. Descriptive statistics (frequency, proportions, mean, standard deviations, etc.) were used to report the study variables. A reverse score was performed for negatively stated statements. For comparisons and running further analysis, the scores for scale variables (attitude, perceived threat, and perceived efficacy) were weighted by multiplying the respective scores by 100, divided by the corresponding maximum scores. Operational definitions were used to guide the analysis of some variables. Bivariate analysis was performed (using independent sample t-test, and Chi-square) to compare changes in weighted means and proportions of exposure, comprehensive knowledge, attitude, perceptions, and practices between baseline and end-line surveys. The changes were labeled as effect sizes (ES). A 95% confidence interval and <0.05 level of significance was used to declare statistically significant differences. Finally, we assessed the relationship of a school-based exposure status and content intensity with observed changes in message acceptance, knowledge, and practices. We used the logistic regression for exposure status and linear regression for content intensity. The findings are presented in text and tables.

#### Ethical approval and consent to participate

The study was reviewed and approved by the Jimma University Institutional Review Board (IRB). Official permission letters to undertake the study were obtained from the concerned bodies at all levels. Respondents were read out loud detailed information about the objectives, potential risks and benefits, rights, and purpose of the study. Informed written consent was obtained from all study participants. A copy of the information sheet having the investigators’ addresses was left with every respondent.

## Results

### Socio-demographic characteristics

A total of 1,521 (baseline = 762 and end-line = 759) households (HHs) participated in the surveys. Overall, 7,785 people lived in sampled HHs. The average family size was 5.1 ± 2.1. The mean age of the respondents was 38.1 ±13.6 years. A majority of respondents were married (87%), Oromo (77%), Muslim (58.5%), and cannot read or write (47.4%). 15.6% of the population was under five years old. The prevalence of pregnancy was 3.1% among women in the reproductive age group. Table 1 presents the details of the socio-demographic characteristics.

**Table 1:** Socio-demographic characteristics of respondents, school-based malaria SBCC target districts, Jimma Zone, Ethiopia, 2017–2019 (baseline, n = 762; end line, n = 759).

Socio-demographic characteristics		Repeated surveys					
		Baseline		End-line		Combined	
		No	%	No	%	No	%
**Districts (Sampled people)**	Botor-Tolay [HH = 239]	699	17.4	606	16.1	1305	16.8
	Nono-Benja [HH = 248]	704	17.5	651	17.3	1355	17.4
	Limmu-Kossa [HH = 264]	589	14.6	615	16.4	1204	15.5
	Gera [HH = 333]	849	21.1	759	20.2	1608	20.7
	Shebe-Sombo [HH = 437]	1184	29.4	1129	30.0	2313	29.7
	Total [HH = 1521]	4025	100	3760	100	7785	100
**Sex of respondent**	Male	378	49.8	439	57.9	817	53.7
	Female	384	50.2	320	42.1	704	46.3
**Sex of HH head**	Male	679	89.2	690	90.9	1369	90.0
	Female	83	10.8	69	9.1	152	10.0
**Marital status**	Married	676	89.2	642	85.0	1318	87.1
	Divorced	26	3.4	31	4.1	57	3.8
	Widowed	41	5.4	29	3.8	70	4.6
	Others	15	2.0	53	7.2	68	4.5
**Education**	Cannot read or write	402	52.8	313	41.2	715	47.0
	Read & write	81	10.6	80	10.6	161	10.6
	Primary school	216	28.3	251	33.1	467	30.7
	Secondary school	60	7.9	106	14.0	166	10.9
	College and above	3	0.4	5	1.1	8	0.5
**Religion**	Muslim	465	61.0	423	55.9	888	58.5
	Orthodox	194	25.5	230	30.4	424	27.9
	Protestant	103	13.5	104	13.7	207	13.6
**Ethnicity**	Oromo	593	77.8	576	76.0	1169	76.9
	Amhara	122	16.0	138	18.2	260	17.1
	Others	45	5.9	54	7.0	99	6.5
**Proportions of**	HHs with school child	535	70.3	458	60.4	986	65.2
	<5 years population	631	15.6	584	15.5	1215	15.6
	*PW/ 15–49 years females	26	2.8	31	3.4	57	3.1

* PW: Pregnant Women

## Changes in community exposure to malaria messages: Reach, repetition, and intensity

### A. Exposure to malaria preventive messages from any source

Table 2 presents the details of the recalls of exposure. The study revealed that households’ exposure to malaria messages over the last 6 months was improved in many ways: reach, repetition, and content intensity. The community reach by malaria messages was improved by ES = 65.7%, p <0.001. There was a significant increase in sources of malaria messages over the intervention period (ES = 7.9%, p <0.001). This refers to the amount of source repetition. Additionally, there was a shift in common sources of malaria messages (t = 9.74, p <0.001). The school community appeared in the top three sources at the end-line compared to the baseline. For example, student visibility as a source of malaria message was increased by ES = 42.0, p = 0.003. The content intensity or exposure to doses of malaria messages from all sources was increased by ES = 11.3%, p <0.001. The specific malaria message that improved the content intensity included giving priority to sleep under ITN, ES = 28.3%; proper use of anti-malarial drugs, ES = 22.2%, how to wash ITN, ES = 16.5%; early treatment-seeking for fever, ES = 15.6%, and IRS spraying, ES = 13.5%, p <0.001.

**Table 2:** Magnitude of changes in community exposure to malaria messages, Jimma Zone, SBCC target districts, 2017–19 (Baseline, n = 762; End-line, n = 759).

Elements of exposure (last 6 months)	Magnitude of exposure				Change	Statistical tests	
	Baseline		End-line		ES		
	No.	%	No.	%	% (95% CI)	t/Chi-square	p-value
**A. Exposure: Reach (rate)**	72	9.4	569	75.1	+65.7 (+54.5,+73.4)	x^2^ = 670.0	<0.001
**Specific sources of exposure**	**n = 504**		**n = 3,963**				
Health Extension Workers (HEWs)	47	65.3	400	70.3	+5.0 (-2.1, +11.3)	x^2^ = 0.763	0.382
School community (student/teachers)	3	4.2	263	46.2	+42.0 (+33.4, +51.2)	x^2^ = 31.96	0.003
Health workers (professionals)	8	11.1	209	36.7	+25.6 (+21.2, +31.3)	x^2^ = 18.74	<0.001
Radio	24	33.3	160	28.1	-5.2 (-7.3, + 2.4)	x^2^ = 0.849	0.357
Health Development Army/neighbors	6	8.4	77	13.5	+5.1 (-8.2, +3.2)	x^2^ = 2.61	0.863
Television (TV)	3	4.2	37	6.5	+2.3 (-2.5, +7.6)	x^2^ = 0.60	0.440
Others*	21	29.2	43	9.0	-20.2 (-28.4, -14.5)	x^2^ = 117.0	<0.001
**B. Exposure: Source repetition** ***	112	22.1	1,189	30.0	+7.9 (+5.1, +12.2)	t = 9.74	<0.001
**Specific message contents of exposure (counts)**	**n = 504**		**n = 3,963**				
Sleeping under ITN-every night	59	81.9	460	80.8	-0.8 (-2.4, +1.9)	x^2^ = 0.42	0.799
Environmental sanitation is preventive	64	88.9	447	78.4	-10.5 (-14.3, -5.8)	x^2^ = 4.31	0.038
Proper use of anti-malarial drugs	10	13.9	206	36.1	+22.2 (+17.6, +28.7)	x^2^ = 4.18	<0.001
Give priority to PWs & <5 (ITN)	5	6.9	200	35.2	+28.3 (+19.5, +37.1)	x^2^ = 23.45	<0.001
Seek treatment for fever early	7	9.7	144	25.3	+15.6 (+11.3, +19.4)	x^2^ = 8.58	0.003
How to wash ITN**	3	4.2	118	20.7	+16.5 (+7.2, 24.9)	x^2^ = 11.43	<0.001
Indoor residual spraying (IRS)**	5	6.9	116	20.4	+13.5 (+6.3, +21.6)	x^2^ = 7.51	<0.001
**C. Exposure: Content intensity** ***	153	31.4	1,691	42.7	+11.3 (+8.9, 15.3)	t = 4.29	<0.001

*Others: religious leaders at mosques, church, and community gatherings; print materials; market places; traditional associations, etc.

**How to wash ITN refers: every 3 months, don't dry in sunlight, don't wash on a hard surface, stitch when torn, etc., and IRS; don't post the wall within 6 months of spray.

^***^Counts of person-to-all sources (for repetition) and person-all specific messages (for content intensity), and ES: Effect Size.

### B. School-based exposure to malaria messages: Reach and intensity

Table 3 presents the details of the recalls of school-based exposure to malaria messages. The study found out a significant increase in the school-specific community reach with malaria messages over the last 6 months (ES = 44.5%, p <0.001). This refers to student engagement in educating the community about malaria that is improved at the end-line compared to baseline. The content intensity of malaria messages delivered to parents through school-aged children showed a significant increase, ES = 19.3%, p <0.001. This means that the diversity of messages the students were transmitting was improved. The specific messages that increased the dosage include proper use of anti-malarial drugs, ES = 38.7%; early treatment-seeking for fever, ES = 30.2%; giving priority to sleep under ITN, ES = 28.8%; and how to wash ITN, ES = 30.9%, p <0.010. We compared the ratio of the content intensity of message exposure via school students to the general sources, compared between the end-line and baseline, which showed a significant increment by, ES = 24.1% at end-line, p = 0.001. This indicates that empowered students could be able to disseminate 24% extra content of malaria messages compared to any other sources in their community.

**Table 3:** Magnitude of changes in community exposure to malaria messages via school-aged children, Jimma Zone, SBCC target districts, 2017–19 (Baseline, n = 762; End-line, n = 759).

Exposure (last 6 months)	Magnitude of exposure				Change	Statistical tests	
	Baseline		End-line		ES		
	No.	%	No.	%	% (95% CI)	t/Chi-square	p-value
**Have a school child**	535	70.3	458	60.4	-9.9 (-13.2, -4.3)	x^2^ = 17.78	<0.001
**Number of school children in the HH**						t = -0.70	0.493
One	189	35.3	155	33.8	+3.5 (-1.3, +7.7)	x^2^ = 3.45	0.112
Two	147	27.5	141	30.8	+3.3 (-2.7, +6.8)	x^2^ = 2.23	0.213
Three	106	20.0	97	21.2	+1.2 (-1.9, +4.2)	x^2^ = 1.04	0.345
Four or higher	93	17.2	65	14.2	-3.0 (-5.2, +1.8)	x^2^ = 1.76	0.327
**A. Exposure: reach via school child**	71	13.3	266	57.8	+44.5 (34.7,51.3)	x^2^ = 218.1	<0.001
**Specific message contents of exposure (counts)**	**n = 568**		**n = 2,128**				
Sleeping under ITN-every night	52	73.2	215	76.2	+3.0 (-1.5, +8.2)	x^2^ = 0.28	0.598
Environmental sanitation is preventive	55	77.5	174	61.7	-15.8 (-20.2, +9.8)	x^2^ = 6.18	0.013
Proper use of anti-malarial drugs	3	4.2	121	42.9	+38.7(+51.4, 23.5)	x^2^ = 32.25	<0.001
Seek treatment for fever early	7	9.9	113	40.1	+30.2 (+21.9, +39.3)	x^2^ = 23.10	<0.001
Give priority to PWs & <5 (ITN)	7	9.9	109	38.7	+28.8 (+22.3, +34.6)	x^2^ = 21.32	<0.001
How to wash ITN**	4	5.6	103	36.5	+30.9 (+25.7, +37.8)	x^2^ = 25.62	<0.001
Indoor residual spraying (IRS)**	1	1.4	91	32.3	+30.9 (+21.5, +41.8)	x^2^ = 28.03	<0.001
Others*	13	18.3	16	5.7	-12.6 (-15.6, -8.6)	x^2^ = 12.01	<0.001
**B. Exposure: Content intensity** ***	142	25.0	942	44.3	+19.3 (+14.3,+25.2)	t = 5.93	<0.001
**Ratio of content: School to general**		79.6		104.0	+24.1 (+17.7, +26.8)	5.25	0.001

*Others: Misconceptions about causes of malaria; ITN with torn don't protect from malaria, etc.

**How to wash ITN refers: every 3 months, don't dry in sunlight, don't wash on a hard surface, and IRS; don't post the wall within 6 months of spray.

^***^Person-to- all specific messages count, and ES: Effect Size

## Changes in knowledge of malaria

Table 4 presents details of the knowledge of malaria. The study showed improvement in comprehensive knowledge about malaria, ES = 14.8%, p <0.001. Mentioning mosquito bite as a cause of malaria grew to 74.0%, ES = 20.8%, p <0.001. Mentioning eating dirty food, cold weather, and an empty stomach as a cause of malaria has significantly reduced. In both surveys, feeling cold, fever, and headache were the three common signs of malaria to mention. Of these, significant improvement was observed in fever (ES = 15.0%). Pregnant women and children < 5 years old were identified as the most vulnerable groups to malaria. Keep the environment clean, sleep under ITN, and using repellent were known more at the end-line compared to baseline. Overall, 47.5% knew about how to care for ITN.

**Table 4:** Community knowledge of malaria, Jimma Zone, SBCC target districts, 2017–19 (Baseline = 762; End-line = 759).

Knowledge about malaria	Magnitude of knowledge				Change	Statistical tests	
	Baseline		End-line		ES		
	No.	%	No.	%	% (95% CI)	Chi-square	p-value
**Ever heard of malaria**	761	99.9	757	99.7	-0.2(-0.4, +0.2)	0.43	0.971
**Comprehensive knowledge**	185	24.3	297	39.1	14.8 (+7.8, 20.1)	38.54	<0.001
**Critical signs of malaria**							
Feeling cold	588	77.3	573	75.6	-1.7 (-2.3, +2.9)	0.59	0.442
Fever	456	59.9	567	74.9	+15.0 (+12.3, 18.8)	38.75	<0.001
Headache	480	63.1	514	67.8	+3.9 (-1.5, +6.6)	3.77	0.052
Nausea/vomiting	219	28.8	293	38.7	+9.9 (+6.4, +12.7)	16.58	<0.001
Loss of appetite	192	25.2	252	33.2	+8.0 (+5.4, 13.3)	11.79	0.001
**Cause of malaria: Mosquito bites**	404	53.2	560	74.0	+20.8 (15.6, +26.8)	70.96	<0.001
**Misconceptions about causes**							
Eating dirty food/drink	340	44.7	170	22.5	-22.2 (-29.1, -16.8)	83.99	<0.001
Cold or changing weather	192	25.2	111	14.7	-10.5 (-16,7, -5.7)	26.40	<0.001
Getting soaked with rain	76	10.0	62	8.2	-1.8 (-4.3, +2.2)	1.50	0.221
Empty stomach	96	12.6	57	7.5	-5.1 (-9.6, -3.4)	10.89	0.001
**How can people protect from malaria**							
Sleep under a mosquito net	429	56.6	597	78.8	+22.2 (+17.0, +27.1)	85.11	<0.001
Fill puddles/stagnant water	625	82.5	342	45.2	-37.3 (-43.2, -31.0)	228.0	<0.001
Keep surroundings clean	45	5.9	417	55.0	+49.1 (+23.7, 61.2)	430.8	<0.001
Indoor Residual Spray	70	9.2	53	7.0	-2.2 (-4.5, +2.5)	2.53	0.111
Using mosquitoes repellent*	-	-	79	10.4	-	-	-
**Who is most at risk of malaria?**							
Pregnant women	582	76.7	586	77.4	+0.7 (-0.6, +1.4)	0.16	0.735
< 5 years children	672	88.5	564	74.4	-13.9 (-18.3, -6.7)	50.18	0.006
Know how to care ITN*^,^ **	-	-	359	47.5	-	-	-

** Multi-dimensional care of ITN; how to wash, how to keep it healthy, and how to repair when torn or has a hole.

* Only end-line data

## Changes in psychological readiness to protect from malaria: Message acceptance

Table 5 presents the details of message acceptance. The surveys indicated a high perceived severity of malaria and the effectiveness of preventive measures. The perceived threat from malaria showed a slight decrease at the end-line compared to baseline (ES = -2.2, p <0.012). On the other hand, the perceived efficacy of protection measures was improved (ES = +2.3, p <0.001). A balance between perceived threat by malaria and the efficacy of advised measures located more people in the danger control zone at the end-line compared to baseline. This improvement in people existing in the danger control zone revealed that there was more psychological readiness towards the uptake of preventive measures. The messages were positively accepted. Over the intervention period, ES = 10% of the community was moved to control the danger of malaria, indicating the magnitude of change in psychological readiness to uptake preventive actions.

**Table 5:** Community malaria message processing and acceptance, Jimma Zone, SBCC target districts, 2017–19 (Baseline = 762, End-line = 759).

Perceptions and acceptance	Weighted means and differences				Change	Statistical tests	
	Baseline		End-line		ES		
	Mean	±SD	Mean	±SD	% (95% CI)	t/Chi-square	p-value
Perceived Susceptibility	50.3	13.4	47.0	11.0	-3.3 (-5.7, -0.9)	t = -5.21	0.01
Perceived Severity	84.4	15.0	83.4	12.3	-1.0 (-3.7, +1.7)	t = 3.21	0.101
**Perceived Threat (PT)**	67.3	16.5	65.1	16.1	-2.2 (-2.8, -1.7)	t = 6.72	0.012
Self-Efficacy	72.9	15.8	81.3	15.7	+8.4 (+8.3, +8.5)	t = 7.97	<0.001
Response Efficacy (attitude)	95.2	3.9	91.4	6.1	-3.8 (-6.0, -1.6)	t = -9.34	0.004
**Perceived Efficacy (PE)**	84.0	13.2	86.3	12.4	+2.3 (+1.7, +3.3)	t = 10.31	<0.001
**Message processing: DV = PE-PT**							
Discriminative Value (DV)	16.8	2.3	21.2	2.6	+4.5 (+4.1, +4.7)	t = 7.25	0.01
Message acceptance*	No.	%	No	%	% (95% CI)		
Fear control (DV≤0)	195	25.8	120	15.8	-10.0 (-16.0,-4.0)	x^2^ = 8.79	0.01
Danger Control (DV>0)	564	74.2	640	84.2	+10.0 (4.0, 16.0)	x^2^ = 8.79	0.01

* The mean and SD are replaced by number and percent, respectively for message acceptance.

## Changes in malaria prevention and control practices

The study found that the proportion of households with at least one ITN grew by 11.5% (p <0.001). The proportion of people who slept under the ITN previous night of surveys was improved (ES = 25.8%, p <0.001). The proportion of people slept under ITN among those who had access was improved by 16.3%, p = 0.01, indicating the magnitude of behavioral failure to use ITN irrespective of access, that was, improved. Environment cleaning practices were assessed only at the end-line; 10.5% of HHs engaged in cleaning their surroundings, at least once per month. At the end-line, more HHs refrained from painting or plastering home within 6 months of spraying (ES = 61.3%, p <0.001). Seeking treatment for fever from any health facility improved by 16.3%. Moreover, timely treatment-seeking (within 24 hours of onset) was improved by 15.5% (25.7% to 41.2%). The experience of taking any drugs for fever increased from 75.1% to 85.2% (ES = 14.1%). Nonetheless, improvement was observed on taking antibiotics (ES = 14.4%, p <0.001). 19.0% (24/126) of fever patients who took any drugs were treated with anti-malarial drugs across the surveys. 41.7% (10/24) received anti-malarial drugs without confirmation through blood testing (no difference between the surveys). Moreover, no significant difference was observed in both drug sharing and adherence. Table 6 refers to the practices.

**Table 6:** Change community malaria preventive and control practices, Jimma Zone, SBCC target districts, 2017–19 (Baseline = 762; End-line = 759).

Preventive and control measures	Magnitudes of the practices				Change	Statistical tests	
	Baseline		End-line		ES		
	No.	%	No.	%	% (95% CI)	Chi-square	p-value
**ITN access and utilization (n = 7,785)**	n = 4,025		n = 3760				
HHs owning at least 1 ITN	572	81.1	703	92.6	+11.5 (8.1,14.9)	x^2^ = 6.61	<0.001
People access to ITN	2,141	53.2	2,955	78.6	25.4 (20.2,30.7)	x^2^ = 9.43	<0.001
People who slept under ITN last night	1,497	37.2	2,369	63.0	25.8 (23.8,27.9)	x^2^ = 23.53	<0.001
People slept under ITN(given access)	1,216	56.8	2,160	73.1	16.3 (8.4, 23.9)	x^2^ = 4.10	0.01
Pregnant women slept under ITN	12	46.2	22	71.0	24.8 (7.3, 43.2)	x^2^ = 4.21	0.01
< 5 years old children slept under ITN	340	53.9	410	70.2	16.3 (4.8, 29.7)	x^2^ = 9.34	0.01
**Environmental manipulation** *			n = 759				
Surrounding**	-	-	80	10.5	-	-	
Compound**	-	-	127	16.7	-	-	
**Healthy handling of IRS (n = 148)**	n = 38		n = 110				
IRS sprayed over the last 12 months	264	34.8	188	24.8	-10.0 (-14.3, -4.7)	x^2^ = 6.12	0.04
Wall painted or plastered (last 12 months)	38	14.4	110	58.5	+44.1 (+18.7, +63.5)	x^2^ = 30.4	0.01
Health plastering of walls after IRS ***	4	10.5	79	71.8	+61.3 (+44.0, 71.1)	x^2^ = 53.56	0.001
**Treatment and drug use practice (n = 165)**	n = 104		n = 61				
Prevalence of febrile illness	104	2.6	61	1.6	-1.0 (-1.5,-0.5)	x^2^ = 4.32	<0.001
Sought treatment for fever (n = 165)	70	67.3	51	83.6	+16.3 (+11.4, +22.7)	x^2^ = 5.23	0.02
Early treatment (within 24 hours)	18	25.7	21	41.2	+15.5 (+5.3, +24.6)	x^2^ = 3.22	0.04
Used any drug for fever	74	71.2	52	85.2	+14.1 (+7.8, +21.1)	x^2^ = 4.23	0.03
**Drugs used for fever (n = 126)**	n = 74		n = 52				
Anti-malarial	14	18.9	10	19.2	+0.3 (-0.7, +0.8)	x^2^ = 0.02	0.97
Anti-pain	31	41.9	23	44.2	-10.5 (-16.7, +2.5)	x^2^ = 0.07	0.79
Anti-biotic +	29	39.2	28	53.6	+14.4 (+9.5, +19.6)	x^2^ = 8.24	<0.001
Blood checked before anti-malarial	4	28.6	6	60.0	+31.2 (-8.6, +52.7)	x^2^ = 2.37	0.124
Anti-malarial drug sharing	4	28.6	5	50.0	+21.4 (-12.5, +54.7)	x^2^ = 3.12	0.273
Adherence to drugs taken for fever ****	55	76.4	35	67.2	-7.1 (-16.9, +4.5)	x^2^ = 0.94	0.332

* Only end-line data.

**HHs engaged in cleaning surrounding at least once per month. **HHs engaged in cleaning their compound from anything suitable for mosquito breeding.

**** No plastering is done within 6 months of the IRS. ****complete, no sharing, no saving, and take anti-malarial drugs without blood checking.

## School-based exposure and relationship with behavior changes over the intervention period

Table 7 presents the analysis of relationships between exposure and key changes. The regression analysis revealed that most of the variables indicated with improvements at the end-line compared to baseline were correlated with the school-based exposure status and intensity of the messages. For example, the odds of community exposure to school-based messages were averagely 9 times higher on the end-line than at baseline. Moreover, ITN utilization and not plastering walls within 6 months of spraying were higher among respondents with school-based exposure to malaria messages compared to households with no school exposure. On the other hand, for a unit increase in school-based message content intensity, there was about 1.8 increases in comprehensive knowledge. A comprehensive knowledge was associated with the message content intensity than mere exposure to school-based communication. Respondents with high comprehensive knowledge had 21% (95% CI: 9.6–32.3%) higher exposure to multiple message sources (repetition). School-based message content intensity was 19.2% higher at the end-line than baseline. Similarly, respondents in danger control averagely had a 7.5% higher weighted score of school-based message content intensity.

**Table 7:** School-based exposure to malaria messages and relationship with key variables of change, Jimma zone, SBCC target districts, 2017–2019, Ethiopia.

**Key change variables**	**School-based exposure to malaria message**		
	School exposure status	Contents intensity	
	Odds ratio	Unstandardized ß coefficient	
	(95% CI)	95% CI	t-test, p-value
Intervention (end-line)	8.9 (6.5,12.2)*	19.2(12.8, 25.5)*	5.93, p <0.001
Comprehensive knowledge	3.3 (0.74, 2.6)	1.8 (1.4, 2.6)*	1.53, p <0.001
Message acceptance	1.1 (0.95,1.3)	7.5 (4.1, 12.7)*	26.18, p <0.004
ITN utilization	1.9 (1.4, 2.4)*	8.2 (2.6, 13.7)*	16.75, p <0.001
ITN care knowledge	1.2 (1.1,1.4)*	24.8 (5.0, 49.1)*	20.1, p = 0.03
Early treatment seeking	1.4 (0.5,3.88)	-5.7 (-20.5,9.8)	-7.44, p = 0.431
Cleaning surrounding	0.7 (0.4,1.3)	2.6 (-8.4, 13.5)	0.46, p = 0.362
Cleaning compound	1.1 (0.6,1.8)	-1.5 (-10.7, 7.7)	-3.23, p = 0.371
IRS healthy handling	3.9 (1.6,9.5)*	6.7 (-9.5, 22.8)	0.83, p = 0.112

*Significant effect

## Discussion

This school-based communication study has produced significant findings on changes in community malaria messages exposure, processing, and actions. RBM recommended that malaria SBCC indicators should enhance community exposure, knowledge, risk perception, and perceived efficacy for malaria preventive actions. The main findings of exposure, knowledge, risk-response appraisals, and practices are discussed as follows. This repeated survey reported a significant change in community exposure to malaria messages from multiple sources (ES = 65%). The amount of change in exposure to the messages from a school-specific source was 44.5%. The exposure to malaria messages included reach, the proportion of exposure over the last 6 months; repetition, the proportion of counts in person-to- all sources of exposure; and intensity, the proportion of counts in person-to-diversity of messages. These elements of exposure were significantly boosted after school-based SBCC intervention. Moreover, school students appeared as one of the top sources of exposure to malaria messages in the post-intervention survey. To the best of our knowledge, reports about community exposure in terms of reach, repetition, and content intensity via school-going children have been limited. The RBM specifies that SBCC for malaria prevention should aim to measure these elements of exposure [19–21]. Perhaps, this study was the first of its kind to produce findings of exposure based on the needed indicators. However, few school-based studies have focused on changes in students' malaria knowledge and parasitemia [61, 62].

In this study, a significant rise (ES = 14.5%) in overall knowledge about malaria and reduction in misconceptions were observed between the two ends. However, even post-intervention knowledge (39.3%) was lower compared to amounts reported in studies in Africa [48–50] and the national elimination plans [12, 13]. Perhaps the variations can be attributed to the following points. First, we measured a comprehensive knowledge most strictly in that we formulated from counts of series of specific knowledge items on causation, commonest symptoms, prevention and treatment methods, and most vulnerable population segments. For example, one study in Equatorial Guinea reported 35% of the combined knowledge score on malaria among rural sub-groups [51]. Our finding reported closer figure of combined knowledge at end-line. Second, many studies reported specific measures of knowledge rather than a comprehensive one, and specific knowledge could expectedly be high [48–50]. For example, even in this study, the specific knowledge that malaria transmits through the bite of infected mosquitoes was as high as 63% at the end-line. Moreover, we strongly argue the measure of malaria knowledge should be a comprehensive one if programs and strategies should initiate sufficient changes in perceptions, attitudes, and practices towards malaria elimination. Furthermore, according to cognitive dissonance theory, when numerous items are used to measure knowledge, the probabilities are high for the facts involved in the scales to initiate cognitive dissonance, create interest in seeking further information about malaria preventive measures towards consonance i.e. change [52].

This study explored how messages about malaria were cognitively and affectively processed by members of the target community in terms of the perceived risk of malaria versus the perceived efficacy of advised preventive practices. Accordingly, this study reported a significant reduction in the perception of vulnerability to malaria. This invulnerability was perhaps justified by a) falling trend in the incidence of malaria and increased knowledge and clearance of misconceptions about causations. Pieces of evidence report malaria incidence have been constantly declining throughout the globe and so do risk perception [7–13, 21, 41, 53]. In this study perceived severity of malaria was consistently high across the repeated surveys, and no significant change was observed. Interestingly, a significant but low reduction in attitude toward preventive actions was revealed even in the presence of improved practices, knowledge, and reduced misconceptions. Although, paths of occurrence were not established in the current article, the decrease in attitude could be explained in the following ways: 1) according to the extended parallel process model, the perceived efficacy of responses or preventive measures i.e. attitude would be processed based on the magnitude of risk perception [at lower perceived risk, there will be lower or no processing of attitude] [47, 53]; 2) potential path of occurrence—may be lower attitude and risk perception followed the improvement in uptake of preventive practices [47, 53,54]; 3) clear up in misconceptions about causations, an impression that the incidence of malaria is experiencing a fall shift [41, 53,54], 4) uptake of preventive practices might have happened through observation or because of a shift in norms (i.e. peripherally) than centrally through beliefs and attitude. According to elaboration the likelihood model, people's attitude or practice may be influenced by some externally observable characteristics like for example how respected people act rather than based on specific message contents [55], 5) may be the observed amount of reduction in attitude is less than a threshold required for withholding preventive practices from happening. This study investigated household malaria message acceptance status (a net balance of messages available in respondents’ mentality either in danger control i.e. accept messages in favor of protection or fear control i.e. ignore messages) by calculating the difference between the perceived efficacy of recommended preventive measures and perceived threat from malaria. The magnitude of people proactively in favor of uptake of preventive-treatment measures (net positive excess) was improved (ES = 10%) over the periods of SBCC intervention. According to the extended parallel process model, this balance was supposed to discriminate between those who uptake preventive practices based on their rationality from those who felt ignorant (net negative excess) even in the presence of adequate preventive or treatment resources because of their emotional impulses of incapability to prevent or control malaria [47].

The current school-based study revealed that the SBCC produced positive changes in practices, especially on ITN usage (ES = 25.8%), priority giving to children <5 years old (ES = 16.3%) and pregnant women (ES = 24.8%), early treatment-seeking for fever (ES = 15.5%), and not plastering or painting walls within 6 months of spraying (ES = 61%). Some studies in Africa reported a sustained uptake of preventive practices-ITNs and IRS in settings with reduced malaria transmission and low-risk perception because a considerable level of threat was present [53,54]. Moreover, pieces of evidence indicated school-based studies produced a change in treatment-seeking and ITN usage and other public health challenges [26–34]. Nonetheless, even the end-line magnitude of ITN usage (63%) and early treatment-seeking (42%) were found to be low compared to the NSMP III plans, that is, 100% suspected cases seek care within 24 hours of onset and at least 80% of people at risk would use ITN [12]. This study revealed a significant reduction (ES = -16.3%) in the behavioral failure of usage of already accessed ITN. This shows there was an improvement in protection mentality that reduced people’s failure to use ITN for reasons other than access. Studies report positive discriminative values to have a high predictive capacity for actual practices [47, 56]. Some studies report that given strongly held protective perceptions and intentions were present, people tend to withstand even the challenges of access [53, 57]. This study observed, even post-intervention, a huge gap (26.9%) of behavioral failure to use ITNs compared to the ENSMP plans III that is aimed to access ITN for 100% of households in their sleeping spaces and 80% of people at risk [12]. Regarding antimalarial drug-related practices, there were no significant changes in sharing and adhering. Nonetheless, the respective magnitudes at the end-line: sharing (7.7%), use of antimalarial drugs without confirmed blood test (41.7%), and adherence (73.3%) were still in the stages needing further improvement compared to national plans-III of 100% [12]. Studies in Africa report that changes in behaviors are complex when deeply rooted in culture, traditional norms, and drug usage is one of them [58,59]. Against the intention of the current SBCC intervention, the use of antibiotics was significantly increased for fever management. Perhaps, such unintended effects were attributed to patients' experience of a frequent negative result from blood tests as a result of a significant decline in the incidence of malaria, and the patients may get treatment for symptoms [59,60].

## Limitations of the study

This study had a strong basis of the fundamentals of multiple theories of communication and behavior change to describe malaria message acceptance and uptake. It also considers RBM behavioral performance indicators required in the era of malaria elimination-eradication plans. Schools and school-based education are currently getting global attention for involvement in public health. For example, school-aged children focused on educational and parasitic clearance intervention studies in Mali suggested a comprehensive school-based control strategy as a feasible and affordable way to address the burden of malaria among school children [61, 62]. However, there are limited studies focused on demonstrating that teaching students in school increase awareness at home that the current study aimed for. Nonetheless, this study was not without limitations. First, there was no control community i.e baseline data were taken as a control for the end-line. The hypothesized effects of the current strategy need further testing through a randomized community trial. Second, this report did not scope to show the mechanisms of occurrences of the changes observed in the community. A recall could be a potential source of bias, especially for exposure. Furthermore, some variables were only measured at the end-line that changes could not be observed.

## Conclusions

This school-based malaria SBCC produced a considerable change in exposure to malaria messages and practices. The introduction of school-based SBCC into the community reshuffled the common sources of and added to the contents of malaria messages. Approaching malaria communication through schools was promising in that it resulted in students’ visibility as common sources of messages and higher penetration with more messages. Despite substantial positive changes in comprehensive knowledge, message acceptance, practices of ITN, timely treatment-seeking for fever, and IRS handling, the amount should still increase to meet national malaria strategic plans and guidelines. The magnitude of environmental cleaning was on stage seeking to undergo massive changes, particularly in mosquito receptive areas. It looks the slight reductions in risk perception and attitude was not beneath the thresholds required for preventive practices. Understanding the paths of occurrences between risk perception, attitude, and practices needs further investigation. The study signals risk appraisal messages are still required for malaria prevention. Overall, school-based SBCC was an effective strategy to expose and advance the community's behaviors toward malaria prevention and control. We recommend national malaria elimination plans and school health promotion programs to embrace school-based SBCC strategy and primary school students as change agents.

## Supporting information

S1 File Information & written consent form-local language version. (DOCX)

S2 File Information sheet and written consent form-English version. (DOCX)

S3 File Information_pre-testing. (DOCX)

S4 File Questionnaires-Afaan Oromo version. (DOCX)

S5 File Questionnaires-English version. (DOCX)
