# Supplementary material for: School-based social and behavior change communication (SBCC) advances community exposure to malaria messages, acceptance, and preventive practices in Ethiopia: A pre-posttest study
Source: PLoS One. 2020 Jun 25;15(6):e0235189. doi: 10.1371/journal.pone.0235189 (PMC7316301; doi:10.1371/journal.pone.0235189)
Supplement: S1 File — (DOCX) [file pone.0235189.s001.docx]

**1.2. Afan Oromo Version: Guca ragaan itti funaanamu qorannoo jalqabaa fi dhumaa**

**Guca odeeffannoo hirmaattota qorannochaaf kennamu**

Akkam bultan (akkam ooltan). Maqaan koo………………,Yunvarsiitii Jimmaa irraan dhufe. Jijjiirama hawaasaa fi amalaa waggoottaan lamaan darban keessaati karaa manneen barnootaa raawwwatamaa turan madaaluu ykn qorachuu irratti qorannoo gaggeessina. Atilleen akka qo’annoo kana kessatti hirmaattu barbaadna; sababin si filanneef odeefanno kayyoo qo’annoo keenyaaf barbaachisaa ta’e nuuf kennita jennee waan yaadneef. Yoo qo’annoo kana irratti hirmaachuuf fedhii qabaatte, gaafiifi deebii itti fufuun gaaffilee gaafii qo’annaa deebisuuf nugargaaran si gaafadha. Keessattuu gaafii waa’ee dammaqiinsa, ilaalchaafi amala ittifayyadama saaphana siree,ka’uumsa,daddarbiinsa, mallattoolee,ittisaa fi yaala busaa ilaallatan si gaafadha.gaafilee qo’annoo keessa jiran kamiifuu yoo deebii kennu hin barbaanne,dhiisuuf mirga qabda.

Saxilamuu fi Mijachuu dhabuu

Odeeffanno tokko tokko namaaf kennuun bicuu namatti toluu dhiisuu danda’a. Yoo gaafiin sitti hin tolle jiraate deebisuu dhiisuu ni dandeessa.

Faayidaa

Hirmaannaan kee odeeffannoo wa’ee beekumsa, ilaalcha fi ittisaa busaa irratti waan nuu laatuuf, yaalii busaa ittisuuf godhamu keessatti ga’ee guddaa ni qaba.

Honnachiftuu

Qo’anno kan keessatti hirmaachuu keetiif wanti siif kennamu hinjiru. Haata’u malee hirmaannaa keetiif baay’ee sigalateeffanna.

Icitii

Odeefannoon qo’annoo kanaaf sasaabnu iccitiin eeggama. Bar-gaaffii kana keessatti tarii maqaan kee yookiin maatiin kee yoo caqafame ta’ee odeefannoo kana sassabuuf akka haalla nuuf mijjessu qofaaf kan itti fayyadamnu ta’a. Qo’annoof yoo fayyadamnu, maqaan kee irraa haqamee laakkofsa koodii dhunfaan adda baha. Odeefannoon maqaa kee qabu saamsamee kan taa’uuf qo’atan ala nama biraatti hinargisiifamu.

Fedha hirmaachuu

Yoo fedha hin qabaannee qorannoo kana keessatti hirmaachuu dhiisuu ni dandeessa. Hirmaachuu diduun kee fulduratti dhaabata fayyaatti ykn iddoo kamittuu yaalamuu iiratti haala kamiinuu si miidhuu hin danda’u. yoo feete yeroo kamiyyuu gaafiif deebii kana irratti hirmaachuu dhiisuu ni dandeessa, akka hirmaataa tokkotti osoo mirga kee kamiyyuu hin dhabin.

Yoo gaaffii qabaate namoota armaan gaditti maqaan isaanii caqafame miseense garee qorannochaa waan ta’aniif bilbila isaanitti fayyadamuun gaafachuu ni dandeessu.

1. Yohaannis Kabbadaa (Moobayilii:+251913232040):

2. Doctor. Zewdie Birhanu (Moobayilii: +251917025852): gorsa duree projectichaa

3. Prof. Morankar (Mobile:+251917763778): Gorsa itti aanaa projektichaa

Qorannocharratti hirmaachuuf fedha qabdaa? Eeyee _____Lakki _____.

Eeyyee yoo jedhe, odeeffanno guucaa labsii feedhaa irra jiruu dubbisuun mallattoo kee kaa’i.

**Guucaa waliigaltee barreefamaa hirmaannaa hirmaatotaa qorannochaa**

Kanneen armaan gadiitti caqafaman hubadheera:

- Qorannoon kun waa’ee beekumsaa, ilaalcha fi amala/gochalee dhibee busaa ilaalchisee hawaasa kessa jiran fi ergaalee karaa Yuunivarsiitii Jimmaan qindeefamuun dhibee busaan walqabatanii karaa manneen barnootas wagoottan darbaan lamaaf dabarfamanitti saaxilamuu kan sakkata’u ta’uu
- Qorannoon kun gaafiilee waa’ee dammaqiinsa, ilaalchaafi amala ittifayyadama saaphana siree/qoricha busaa/yaala busaa/biifaa keemikalaa/qulqulina naannoo, ka’uumsa,daddarbiinsa, mallattoolee,ittisaa fi yaala busaa ilaallatan kan gaafatu ta’uu.
- Yoo gaafiin natti hin tolle jiraate deebisuu dhiisuu kan danda’u ta’uu.
- Qarshiis ta’e honnachiftuun naaf kennamu kan hin jire ta’uu
- Odeeffaannoo sassabamu icitiin kan qabamuu fi adda baaftuun reportiif kan hin fayyadine ta’uu
- Qorannoon kun itti fayyadama tajaajila hawaasa fi fayyaa ilaalchisee miidhaa adda kan hin qabne ta’uu fi guutummaa guutuutti fedharatti kan hundaa’e ta’uu.

Labsii fedhaa

Odeeffannon armaan olii naaf dubbifameera,hubadheeras. Qorannoon kunis waa’ee beekumsa, hubannoo, ilaalcha fi gochaalee ittisa busaa kan ilaalchisu ta’uu isaa hubadheera. Qorannoo kana irratti hirmaachuun fedhakoo irratti kan hundaa’eefi odeeffannon ani kennu icitiin kan eegame ta’uu isaa hubaadheera. Kanaaf,

Qoranoo kana keessatti hirmaatuuf fedha qabdaa?

Eeyye ________ lakki_______[hirmaataa galateefadhuu gaafannoo guuti].

Maqaa fi mallatoo hirmaataa: Maqaa_____________Guyyaa _________Mallattoo___________

Maqaaf malattoo raga sasaabaa/duu: Maqaa__________Guyyaa__________Mallattoo_____________
