# Supplementary material for: School-based social and behavior change communication (SBCC) advances community exposure to malaria messages, acceptance, and preventive practices in Ethiopia: A pre-posttest study
Source: PLoS One. 2020 Jun 25;15(6):e0235189. doi: 10.1371/journal.pone.0235189 (PMC7316301; doi:10.1371/journal.pone.0235189)
Supplement: S2 File — (DOCX) [file pone.0235189.s002.docx]

**Annexes**

1. **Quantitative: Questionnaire**

**1.1. English version Questionnaire: Data Collection Form Baseline and endline**

**Information sheet form for respondent households**

Good morning (Good afternoon/evening). My name is ……………., I am from Jimma University. We are doing a study to evaluate a social and behavioral change communication interventions through schools and religious leaders, that has been implemented during the past two/three years. We want you participate in this study; we have chosen you because we think you would be in a position to provide with us relevant information about study objectives. If you are willing to participate in the study, I will proceed with the interview and administer questions that help to answer the study questions. Specifically, I will forward questions concerning your awareness, attitude and behaviors concerning use of bed nets, the cause, transmission, symptoms, prevention and treatment of malaria. If you do not wish to answer any of the questions included in the study, you do have the right not to answer any question.

Risks and Discomfort

There might be slight discomfort to share some personal information. However, we do not wish this to happen and you may refuse to answer any of the questions if you feel uncomfortable.

Benefits

Although there will be no direct benefits to you at this moment, your participation will help us to find out more about awareness about malaria and use of ITN, treatment seeking and anti-malarial drug use, in the community and the need of intervention about proper malaria control practices which we think will reduce the burden of malaria in your community. The study also might help sick persons to get early treatment by giving advises and referral.

Incentives

You will not be provided any incentive for your participation in the study. However, we will gratefully acknowledge your participation.

Confidentiality

The information that we collect in this study will be kept confidential. When using the information for research purpose, your name will be removed and it will only be identified by code number. The information containing your name will be kept under lock and will not be divulged to anyone except the investigators.

Right to refuse or withdraw

You do not have to take part in this research if you do not wish to do so, and refusing to participate will not affect your future treatment at the health facility or elsewhere in any way. You may stop participating in the interview at any time that you wish without losing any of your rights as a participant.

If you have any question about this study: you can contant the following persons who are team in this project

1. Yohannes Kebede (Mobile:+251913232040):
2. Dr. Zewdie Birhanu (Mobile: +251917025852): primary advisor of this project
3. Prof. Morankar (Mobile:+251917763778): Secondary advisor of this project

Are you willing to take part in the study? Yes _____No_____.

If yes, proceed to reading the information the consent and then fill the form**.**

**Written consent form for household survey**

I understood that:

- This study is aimed to assess community perceptions, practices and exposure to a school based behavioral change communication interventions through schools that has been implemented over last two/three years in Jimma zone by Jimma University.
- This study is going to ask me questions concerning awareness, attitude about cause, transmission, symptoms, prevention and treatment of malaria and behaviors relating to bed nets, treatment of fever, ant-malarial drug use, IRS acceptance and eviromental cleaning the.
- If I do not wish to answer any of the questions, I do have the right not to answer any question.
- I I will not be provided any incentive for my participation
- The information collected will be kept confidential and identifiers will not be reported.
- This study has no risk on my future of health and social services. Rather my participation in this study is relevant to learn the effect of the project, promote effective strategy and willful

Declaration of the Volunteer

Are you willing to participate in this study?

Yes ________ No_______[thank the participant and document the questionnaire]

Name and signature of the respondent:

Name__________Date __________Signature_____________

Name and signature of data collector:

Name__________Date __________Signature_____________
