# Supplementary material for: School-based social and behavior change communication (SBCC) advances community exposure to malaria messages, acceptance, and preventive practices in Ethiopia: A pre-posttest study
Source: PLoS One. 2020 Jun 25;15(6):e0235189. doi: 10.1371/journal.pone.0235189 (PMC7316301; doi:10.1371/journal.pone.0235189)
Supplement: S3 File — (DOCX) [file pone.0235189.s003.docx]

**Information about pre-testing of the survey questionnaire**

The following points provide the details about pre-testing of the questionnaire

1. The pretesting was conducted in similar settings located in the Jimma Zone, but which were not included in the actual settings where the survey took place: The sites include- two villages in Serbo and Dedo districts.
2. The intention of the pretesting was multiple- to ensure the questions are understandable (length, word selection, etc)
3. To improve the understandability of the tools we accomplished the following activities
   1. Discussed with the data enumerators during the training sessions for clarity and communality across the data collectors.
   2. Role-play was demonstrated by the enumerators pair by pair, and the sub-groups have commented each other taking different scenario about the potential experiences they may face in community
   3. While on the training, we send out trainees for one day pretesting in similar communities
   4. On the next day we discussed on the comments , experiences and procedures to follow while asking and responding during interview
   5. Based on pretest finding, the data collectors were received to follow similar instructions
4. Based on the pretest findings we made the following improvement to the questionnaire:
   1. As much as possible the length of the questions were made shorter
   2. Instructions for how to ask and responded were added or improved to every important sections of the questionnaire
   3. We inserted some questions
   4. Procedures of probing and checking some behaviors like ITN utilization and drug-adherence
