# Supplementary material for: School-based social and behavior change communication (SBCC) advances community exposure to malaria messages, acceptance, and preventive practices in Ethiopia: A pre-posttest study
Source: PLoS One. 2020 Jun 25;15(6):e0235189. doi: 10.1371/journal.pone.0235189 (PMC7316301; doi:10.1371/journal.pone.0235189)
Supplement: S4 File — (DOCX) [file pone.0235189.s004.docx]

**Gaaffilee bar-gaaffii mana-manaa irra deddeebiin funaanamu**

Beeekkumsa, ilaalacha, amantaa, amala waa`ee busaa fi mudannoo ergaalee busaa ilaalan.

| Waliigaltee af-gaafannoo maatiitiif qophaa’e dubbisi | | | | | | | | | | | | | | | | | | | | | | | | | | | |  |
| --- | --- | --- | --- | --- | --- | --- | --- | --- | --- | --- | --- | --- | --- | --- | --- | --- | --- | --- | --- | --- | --- | --- | --- | --- | --- | --- | --- | --- |
| Deeb-kennaan hirmaachuuf hayyamamoodha... 1  🡫Bargaaffii | | | | | | | | | Deeb-kennaa gaafatamuuf hayyamamoo miti🡪2 -------dhaabi | | | | | | | | | | | | | | | | | | |  |
|  | Identifiers | | | | | | | | | | | | | | | | | | | | | | | | | | |  |
|  | Qorannoon kun yoom rawwatamee? 1: Dursa 2: Dhuma (hordoffii) | | | | | | | | | | | | | | | | | | | | | | | | | | |  |
|  | Maqaa Aanaa | | | | | | ______________________ | | | | | | | | | | | | | | | | | | | | |  |
|  | Olka`iinsa gidduu galeessa gandichaa | | | | | |  | | | | | | | | | | | | | | | | | | | | |  |
|  | Maqaa Gandaa | | | | | | ______________________ | | | | | | | | | | | | | | | | | | | | |  |
|  | Maqaa duree maatii | | | | | | ______________________ | | | | | | | | | | | | | | | | | | | | |  |
|  | Zoonii/ garee | | | | | | ______________________ | | | | | | | | | | | | | | | | | | | | |  |
|  | Iddoo jireenyaa | | | | | | 1. Magaalaa 2. Baadiyyaa | | | | | | | | | | | | | | | | | | | | |  |
| 07 | Koodii Bargaaffichaa | | | | | | DD/KK/HH/IN | | | | | | | | | | | | | | | | | | | | |  |
|  | Respondents background | | | | | | | | | | | | | | | | | | | | | | | | | | |  |
| Q1 | Umuriin kee meeqa? | | | | | | _______[waggaadhan] | | | | | | | | | | | | | | | | | | | | |  |
| Q2 | Saala | | | | | | 1. Dhiira 2. Dhalaa | | | | | | | | | | | | | | | | | | | | |  |
| Q3 | Saala dureen maatii? | | | | | | 1. Dhiira 2.Dhalaa | | | | | | | | | | | | | | | | | | | | |  |
| Q4 | Haala gaa`ilaa | | | | | | 1. Fuudhee/heerumtee kan waliin jiraatu/jiraattu 2. Fudhee/heerumtee-gargar kan jiraatu/jiraattu 3. Kan hiike/hiikte 4. Kan irraa du’e/jalaa duute 5. Kan biroo___________ | | | | | | | | | | | | | | | | | | | | |  |
| Q5 | Abbaan manaakee haadha manaa meeqaa qaba? | | | | | | ______________________ | | | | | | | | | | | | | | | | | | | | |  |
| Q6 | Sadarkaa barnootaa | | | | | | 1. Dubbisuu fi barreessu hin danda’u 2. Dubbisuu fi barreessu ni danda’a garuu baruumsa idilee hin baranne 3. Kutaa baratee xumure_______ | | | | | | | | | | | | | | | | | | | | |  |
| Q7 | Sadarkaa barumsaa haadha manaa/abbaa warraa, yoo lubbun jiraate/tte | | | | | | 1. Dubbisuu fi barreesuu hin danda’u/eessu 2. Dubbisuu fi barreessu ni danda’a garuu baruumsa idilee hin baranne 3. Kutaa oli aanaa baratee xumure__________________ 4. hin ilaallatu | | | | | | | | | | | | | | | | | | | | |  |
| Q8 | Amantaa | | | | | | 1. Islaama 2. Ortodoksii 3. Piroteestanti 4. kan biroo [ibsi]________ | | | | | | | | | | | | | | | | | | | | |  |
| Q9 | Saba | | | | | | 1. Oromo 2. Amaara 3. Yam 4. Daawuroo 5. Kan biroo [ibsi]__________ | | | | | | | | | | | | | | | | | | | | |  |
| Q10 | Haala hojii | | | | | | 1. Qonnaan bulaa/tuu 2. Daldalaa/tuu 3. Hojjetaa/ttu mootummaa 4. Hojii dhuunfaa 5. Kan biroo [ibsi]_________ | | | | | | | | | | | | | | | | | | | | |  |
| Q11 | Baayina miseensa maatii [miseensa dhaabbataa= < Ji’a 6 kan maatiin waliin jiru] | | | | | | _____________________________ | | | | | | | | | | | | | | | | | | | | |  |
|  | Gaaffile beekkumsaa/Knowledge | | | | | | | | | | | | | | | | | | | | | | | | | | |  |
| Q12 | Waa’ee dukkuba busaa ykn woba ykn Busaa jedhamuu dhaggeessees beektaa? | | | | | | 1. Eeyyee 2. Lakki | | | | | | | | | | | | | | | | | | | | |  |
| Q13 | Mallatoolee dhukuba busaa isaan gurgudda ta’an natti himuu dandeessa?  Filannoolee hin dubbisin  DEEBIIN HEDDU KENNUUN NIDANDA’AMA  Altokko irra deebi’ii ‘’wanti biroo kan hafe jiraa?’’ Jedhii gaafadhu | | | | | | 1. Qaama gubaa 2. Qorrisiisuu/qorri namatti dhaga`amu 3. Mataa bowwoo 4. Olguuruu fi hooqqisuu 5. Garaa kaasaa 6. Joonjessuu 7. Fedhii nyaataa dhabuu 8. Buusaa caccabsuu 9. Addachuu ijaa 10. Dadhabbii qaamaa 11. Kan biroo (ibsi)_________ 12. Hin beeku | | | | | | | | | | | | | | | | | | | | |  |
| Q14 | Akka yaada keetti dhukkuba buusaa maaltu fida?  Filannoolee hin dubbisin  DEEBIIN HEDDU KENNUUN NI DANDA’AMA  Altokko irra deebi’ii ‘’wanti biroo kan hafe jiraa?’’ Jedhii gaafadhu | | | | | | 1. Bookee buusan iddamuu 2. Boqqoolloo nyaachuu 3. Shonkoora/xinqishaa nyaachuu 4. Beelaan (garaa duwwaa) 5. Nyaata/bishaan qulqulluu hin ta`in nyaachuu/dhuguun 6. Rooba/bokkaan nama tortorsuun/dhaanuun 7. Qorra/ jijjiirama qilleeensaan 8. Mora/tolchaan 9. Kan biroo (ibsi)________ 10. Hin beeku | | | | | | | | | | | | | | | | | | | | |  |
| Q15 | Maatin tokko akkamitti busaa irraa of eeguu danda’a? | | | | | | 1. Saaphana siree jala rafuun 2. Qoricha bookee busaa ari’u fayyadamuun 3. Farra ilbiisotaa manatti biifuun 4. Naannoo manaa qulqullinaan qabuun 5. Iddoo bishaan itti kuufamu guutuun ykn bishaan ciisaa qoorsuun 6. Rooba/bokkaan of tortorsuu/keessa deemuu dhiisuu 7. Kan biroo (ibsi)________ 8. Hin beeku | | | | | | | | | | | | | | | | | | | | |  |
| Q16 | Akka yaada keetti, dhukkubni busaa irra caala eenyuun hubuu/ miidhuu danda’a?    Filannoo dubbisiif, filannoo tarreeffame keessa lama akka filatan godhi | | | | | | 1. Dhiira ga`eessa 2. Dubartii ga`eettii 3. Haadholii ulfaa 4. Daa`ima waggaa jahaa 5. Daa`ima/mucaa waggaa sadii 6. Hin beeku | | | | | | | | | | | | | | | | | | | | |  |
|  | Amaloota bookee busaa: bakka jireenya, ciniinnaa fi of-eeggannoo (Mosquito behavior) | | | | | | | | | | | | | | | | | | | | | | | | | | | |
|  | Bookeen busaa eessatti wal-horu  Filannoota hin dubbisin (Kan biraa jedhii gaaffu)  DEEBII HEDDU KENNUUN NIDANDA’AMA | | | | | | 1. Lafa biishaan ciise/kuufame jiru 2. Biqiltoota bishaan haammatan (fkn: warqee) 3. Qodaa cabaa (fkn: xuwwee) bishaan kuuse 4. Lafaa caffa’aa fi raaree qabu 5. Kan biro (ibsi)________________________ 6. Hin beeku | | | | | | | | | | | | | | | | | | | | |  |
|  | Bookeen busaa eessa jiraatu?  Filannoota hin dubbisin (Kan biraa jedhii gaaffu)  DEEBII HEDDU KENNUUN NIDANDA’AMA | | | | | | 1. Dawoo/golgee/dhooha manaa jala/keessa 2. Bakkaa biishaan xixiqqaan ciisu/kuufame 3. Margaa/balaa boqqolloo fi hophoo warqee 4. Qodaa cabaa (xuwwee fi k.k.f) bishaan kuuse 5. Lafaa caffa’aa 6. Kan biro (ibsi)________________________ 7. Hin beeku | | | | | | | | | | | | | | | | | | | | |  |
|  | Bookeen busaa fageenyaa dheeraa deemuun gara mana keenyaa dhufamii nu hidduu danda’u! | | | | | | 1. Dhugaa 2. Soba 3. Hin beeku | | | | | | | | | | | | | | | | | | | | |  |
|  | Bookeen busaa sa’aa kam keessa nama hiddu?  Filannoo dubbisiif, kan caala sirri ta’e filachiisi | | | | | | 1. Dimimmisa (sa’aa 12) hanga baarii (sa’aa 11) 2. Guyyaa kessa 3. Halkan yeroo ciisaa (sa’aa 3) booda | | | | | | | | | | | | | | | | | | | | |  |
|  | Bookeen busaa eessatti nama hiddu? | | | | | | 1. Mana keessatti 2. Manaa alatti 3. Hin beeku | | | | | | | | | | | | | | | | | | | | |  |
|  | Of-eeggannoon bookee busaan hiddamuu dhorkuuf godhamu maal fa’a?  Filannoota hin dubbisin (Kan biraa jedhii gaaffu)  DEEBII HEDDU KENNUUN NIDANDA’AMA | | | | | | 1. Dimimmisa dura mana cuufuu (sa’aa 11 booda) 2. Dafanii manatti galuu 3. Yeroo dimimisaa’ee eegalee agobara hidhuu (yeroo ciisichaan dura) 4. Yeroo hunda agoobara keessa buluu 5. Bakka wal-hormaata bookee busaa balleessuu 6. Kan biro,__________________________ 7. Hin beekuu | | | | | | | | | | | | | | | | | | | | |  |
|  | Ownership and use of ITNs : Q18 eegalee- | | | | | | | | | | | | | | | | | | | | | | |  | | | | |
| Q17 | Saaphana siree/Hagoobara kan yeroo raftan itti fayyadamtan manaa qabduu? | | | | | 1. Eeyye  2.Lakki | | | | | | | | | | | | | | | | | | Lakki → gara Q23 tti tari | | | | |
| Q18 | Saaphan siree meeqa manaa qabdu? | | | | | ________[baayina saaphana siree] | | | | | | | | | | | | | | | | | |  | | | | |
| Q19 | Saaphana siree (SS) mana keessa jiru akka sitti agarsiisan gaafadhuu. Gabatee kanatti aanu tokkoon tokkoon saaphana sireef guuti. Yoo saaphana torbaa ol ta’e, sarara gadee itti dabalii fayyadami.  Saaphana siree ijaan agartee jirtaa?   1. Kan ilaalame 2. Kan hin ilaalame | | | | | ITN1 | | | | | ITN2 | | | ITN3 | | | ITN4 | | | ITN 5 | | | | ITN6 | | ITN7 | | |
|  |  |  |  |  |  |  | | | | |  | | |  | | |  | | |  | | | |  | |  | | |
| Q20.1 | Saapahanni sirichaa uraa/qaawwa bookee seensisu qabaa ykn tarsa’ee jiraa? (ilaali)   1. Eeyyee 2. Lakki | | | | | ITN1 | | | | | ITN2 | | | ITN3 | | | ITN4 | | | ITN5 | | | | ITN6 | | ITN7 | | |
|  |  |  |  |  |  |  | | | | |  | | |  | | |  | | |  | | | |  | |  | | |
| Q20.2 | Saapahanni sirichaa ji’a sadii kana keessa miicameraa? 1. Eeyee 2. Lakki | | | | |  | | | | |  | | |  | | |  | | |  | | | |  | |  | | |
| Q21 | Saapahanni sirichaa haala akkamii irra jira? [ilaali]   1. Siree/afata gubbaa(irraa) jira 2. Maramee taa’e/kaawwame/ (e.g: saanduqa) 3. Dhimma/Faayidaa biraaatiif oole jira | | | | |  | | | | |  | | |  | | |  | | |  | | | |  | |  | | |
| Q22 | Erga saaphana siree bookee busaa kana argattanii hangam ture? [ji’aan kaa’i] | | | | |  | | | | |  | | |  | | |  | | |  | | | |  | |  | | |
|  | Utilization of ITNs | | | | | | | | | | | | | | | | | | | | | | | | | | | |
| Q23 | Odeeffannoo muraasa waa’ee namoota mana kee keessa jiraachaa jiranii sigaafachuun fedha. Jalqaba maqaa, umrii, saala fi hariiroo/walitti dhufeenya miseensota maatii hundaa naatti himtu? Sana booda saaphana siree bookee busaa eenyuu fi akkamitti akka itti fayyadaman sigaafadha. Quxusuu irraa eegali daa`imman dabalatee.  (Galmeessi, maatii saapahan siree hin qabnes dabalatee) | | | | | | | | | | | | | | | | | | | | | | | | | | | |
| Gaaffilee kanatti aananii jiraniif deebii isaanii gabatee keessatti guuti | | | | | | | | | | | | | | | | | | | | | | | | | | | | |
| Koodii  [DD/KK/HH/IN/##] | | Maqaa | Umurii  (waggaa) | | Halkan darbe mana bule/te  Eeyee=1  Lakki=0 | | | | | saala  Dhiira=1  Dhalaa=2 | | | | | Ulfa  Eeyyee=1  Lakki=0  [yoo umuriin ishee waggaa 15-49 ta`e ) | | | | | | Halkan darbe saapahana siree kana keessa raftanii turtani?  Eeyyee=1  Lakkii=0 | | | | | | | |
|  | |  |  |  |  | | | | |  | | | | |  | | | | | |  | | | | | | | |
|  | |  |  |  |  | | | | |  | | | | |  | | | | | |  | | | | | | | |
|  | |  |  |  |  | | | | |  | | | | |  | | | | | |  | | | | | | | |
|  | |  |  |  |  | | | | |  | | | | |  | | | | | |  | | | | | | | |
|  | |  |  |  |  | | | | |  | | | | |  | | | | | |  | | | | | | | |
|  | |  |  |  |  | | | | |  | | | | |  | | | | | |  | | | | | | | |
|  | |  |  |  |  | | | | |  | | | | |  | | | | | |  | | | | | | | |
|  | |  |  |  |  | | | | |  | | | | |  | | | | | |  | | | | | | | |
|  | |  |  |  |  | | | | |  | | | | |  | | | | | |  | | | | | | | |
|  | |  |  |  |  | | | | |  | | | | |  | | | | | |  | | | | | | | |
|  | |  |  |  |  | | | | |  | | | | |  | | | | | |  | | | | | | | |
|  | |  |  |  |  | | | | |  | | | | |  | | | | | |  | | | | | | | |
|  | |  |  |  |  | | | | |  | | | | |  | | | | | |  | | | | | | | |
|  | |  |  |  |  | | | | |  | | | | |  | | | | | |  | | | | | | | |
| Q23.B) Mana kana keesa bakka ciisichaa (siree fi kutaan adda ba’e dirqama miti) meeqatu jira?_________(## kaa’i) | | | | | | | | | | | | | | | | | | | | | | | | | | | | |
| Q23 C) Halkan darbe, eenyuufaatu bakka ciisichaa kanneenirra waliin bule (> 4 yoo ta’e cinatti sararii guuti)   \| *Bakka ciisichaaf gaafadhuu* \| Ciisicha-1 \| Ciisicha-2 \| Ciisicha-3 \| Ciisicha-4 \| \| --- \| --- \| --- \| --- \| --- \| \| 1. Baayina (##) \| ____________ \| ___________ \| ____________ \| ____________ \| \| 1. Saala (##) \| Dhi___Dha___ \| Dhi____Dha___ \| Dhi_____Dha___ \| Dhi____Dha___ \| \| 1. Dubartii ulfaa \| _______ \| ____________ \| ____________ \| ___________ \| \| 1. Daa’ima umurii <5 \| _______ \| ____________ \| ____________ \| ___________ \| \| 1. Agoobara(Qaba=1,Hinqabu=0) \| _______ \| ____________ \| ____________ \| ___________ \| | | | | | | | | | | | | | | | | | | | | | | | | | | | | |
| Q23D | Beekumsa waa’ee kunuunsa Agoobaraa (ITNs net care knowledge) | | | | | | | | | | | | | | | | | | | | | | | | | | | |
|  | Miiccaa agoobaraa ilaalchisee gochaaleen rawwatamuu hin qabnee maal fa’aa dha? | | | | | | | 1. Saamunaa qorichumaa qabuun hin miiccamu 2. Dhagaa/kattaa irratti hin miicamu 3. Burushiin hin miiccamu 4. Dhuubame hin bulchamu 5. Kallattiin ifa aduun hin qoorfamu 6. Ji’a 3’n keessatti hin miiccamu 7. Kan biro______________ 8. Hin beeku 9. Agoobarri miiccamuu hin qabu | | | | | | | | | | | | | | | | | | |  | |
|  | Agoobarri gaafa miiccamu keemikalli farra ilbiisummaa isaa irra bada | | | | | | | 1. Dhugaa 2. Soba 3. Hin beeku | | | | | | | | | | | | | | | | | | |  | |
|  | Agoobarri akka yeroo malee citee/uratee hin badneef maaltu godhama? | | | | | | | 1. Guyya guyyaa maranii fannisuu 2. Yeroo itti seenanu suutan seenuu, 3. Garmalee osoo hin harkisin fannisuu. 4. Guyyaa guyyaam hordofuu 5. Daa’imni akka keessa hin taphane 6. Kan biro____________ 7. Hin beeku | | | | | | | | | | | | | | | | | | |  | |
|  | Agoobarri yoo citaa ykn urataa qabaatee akkamitti sirreefannee itti fayyadamna? | | | | | | | 1. Lilmoo fi kirriin hodhuun 2. Uraa isaa meeshaa biraan duuchuun 3. Erbee irratti maxxansuun 4. Kan biro____________________ 5. Hin beeku | | | | | | | | | | | | | | | | | | |  | |
|  | Care/treatment seeking practice | | | | | | | | | | | | | | | | | | | | | | | | | | | |
| Q24 | Torbaan lamaan darban keessa, maatii keessan keessaa namni qaama gubaa dhukkubsate jiraa? | | | | | | | | | | | | | | | | | | 1. Eeyye 2. Lakki | | | | | | Lakki→garQ37 tti tari | | | |
| Q25 | Eeyyee yoo ta’e, gabatee kanatti aanu guuti. Nama afurii ol yoo ta’an, gaafaannoo biraa fayyadami | | | | | | | | | | | | | | | | | |  | | | | | | | | | |
| Q25 |  | | | | | | | | | | | | | | | Nam 1 | | | Nam2 | | | Nam3 | | | | Nam4 | | |
|  | Koodii | | | | | | | | | | | | | | |  | | |  | | |  | | | |  | | |
|  | Umurii | | | | | | | | | | | | | | |  | | |  | | |  | | | |  | | |
|  | Saala | | | | | | | | | | | | | | |  | | |  | | |  | | | |  | | |
| Q26 | Qaama gubaan kun guyyaa meeqa dura jalqabe? | | | | | | | | | | | | | | | Nam-1 | | | Nam2 | | | Nam 3 | | | | Nam4 | | |
|  |  |  |  |  |  |  |  |  |  |  |  |  |  |  |  |  | | |  | | |  | | | |  | | |
| Q27 | Ammayyuu (maqaa) qaama gubaatin dhukkubsachaa jiraa/tii?   1. Eeyye 2. Lakki | | | | | | | | | | | | | | |  | | |  | | |  | | | |  | | |
| Q28 | Qaama gubaa kanaaf gorsa yookiin yaala/wal`aansa iddoo kamiiyyuu haa ta’u argatee/ttee turte?   1. Eeyyee 2. Lakki | | | | | | | | | | | | | | |  | | |  | | |  | | | |  | | |
| Q29 | Yaala yookiin gorsa eessa argatte?   1. Kellaa fayyaa 2. Buufata fayyaa 3. Hospitaala 4. Faarmaasii/ mana qorichaa 5. Kilinika dhuunfaa 6. Kan biroo[ibsi]_____   Iddoo biroo jiraa?  IDDO/MADDA EERAME HUNDAA GALMEESSI | | | | | | | | | | | | | | |  | | |  | | |  | | | |  | | |
| Q30 | Eega qaama gubaan jalqabee(Maqaa) guyyoota meeqaa booda jalqabaaf kan yaala yookiin gorsa barbaachaaf deeme/te?  Guyyaadhan | | | | | | | | | | | | | | |  | | |  | | |  | | | |  | | |
| Q31 | Yeroo kamiyyuu haa ta’u tibba dhukkubaa sana, qaama gubaatif qoricha kamiyyuu fudhatee?   1. Eeyyee 2. Lakki | | | | | | | | | | | | | | |  | | |  | | |  | | | |  | | |
| Q32 | Qoricha kam fudhate?  Qoricha biraa kamiyyuu haa ta’u?  Hin beeku yoo ta’e qoricha itti agarsiisi   1. Coartem 2. Chloroquine 3. quinine 4. Percetamol 5. Ibuprofen 6. Kan biroo_____ 7. Hin beeku   KAN EERAME HUNDAA TOKKO TOKKO NAMAAF GALMEESSI | | | | | | | | | | | | | | |  | | |  | | |  | | | |  | | |
| Q33 | Qoricha FARRA BUSAA manaa qabda moo iddo biraatii argatte?  YOO EDDOO BIRAA IRRAA TA’E, MADDA ISAA GAAFADHU: qoricha kana eessaa aragatte? [qoricha FARRA BUSAA qofaaf gaafadhu]   1. Manaa qaba 2. Kellaa fayyaa 3. Buufata fayyaa 4. Kilinika dhuunfaa 5. Suuqii 6. Kan biraa_____ 7. Hin beeku | | | | | | | | | | | | | | |  | | |  | | |  | | | |  | | |
| Q34 | Yeroo ammaa kana (maqaa waami) qoricha fudhachaa jira/jirtii?   1. Eeyyee- fudhataa jira/jirti 2. Eeyye-xumuree jira/jirti 3. Addaan kutte/kute 4. Qoricha hin fayyadamne | | | | | | | | | | | | | | |  | | |  | | |  | | | |  | | |
| Q35 | Qoricha kee nama birootiif qooddee beekta?   1. Eeyye 2. Lakki 3. Qoricha kamiyyuu hin fudhanne | | | | | | | | | | | | | | | Nama1 | | | Nam2 | | | Nama3 | | | | Nam4 | | |
|  |  |  |  |  |  |  |  |  |  |  |  |  |  |  |  |  | | |  | | |  | | | |  | | |
| Q36 | Yeroo qaama gubaa sana dhiigni fudhatamee qoratamee, yoo dhaabbata fayyaatti illalame/te?  Was blood taken and checked during fever, if seen at health facility?   1. Eeyye 2. Lakki 3. Dhabbata fayyaa kamiyyuu hin dhaqne | | | | | | | | | | | | | | |  | | |  | | |  | | | |  | | |
|  | Shaakala keemikaala farra bookee buusaa mana keessatti biiifuu fi qe’ee/nannoo qulqulleessuu | | | | | | | | | | | | | | | | | | | | | | | | |  | | |
| Q37 | Ji’oota 12n darban keessatti, namni mana jireenyaa kee keemikaala farra bookee busaa biife jiraa? | | | | | | | | | | | | 1. Eeyye 2. Lakki →gara Q41tti tari 3. Hin beeku→gara Q41tti tari | | | | | | | | | | | | |  | | |
| Q38 | Manichi erga itti biifamee Ji’oota meeqa ta’eera? | | | | | | | | | | | | Ji`a______ | | | | | | | | | | | | |  | | |
| Q39.1 | Ji’oota 12n darban keessatti yeroo kamiyyuu, manni keessan marigamee yookiin halluun dibamee beekaa? | | | | | | | | | | | | 1. Eeyye 3. Hin beeku 2. Lakki | | | | | | | | | | | | |  | | |
| Q39.2 | Erga manni maarigamee/halluufamee ji’oota meeqa ta’eera? | | | | | | | | | | | | Ji`a ______________ | | | | | | | | | | | | | | | |
| Q40.1 | Ji’oota 6’n darban keessa yeroo meeqa naannoo (qe’een alatti) keessan bakka bishaan kuufate irraa qulqulleesitanii jirtu? | | | | | | | | | | | | _____________________(lakkofsaanka’i) | | | | | | | | | | | | | | | |
| Q40.1 | Ji’a 3’n darbe keessa yeroo meeqa qe’ee keessan wantoota bishaan kuusuu danda’an kamirraayyuu qulqullesitanii jirtu? | | | | | | | | | | | | _____________________(lakkoofsaan ka’i) | | | | | | | | | | | | | | | |
| Q40.2 | Qe’ee fi naannoo keessan yeroo qulqullesitan busaa ittisuuf maal maal fa’a irraa qulqulleesitu? ( deebii > 1 ni danda’ama | | | | | | | | | | | | 1. Qodaa caba 3. Sogoo warqee 5. Marga 2. Laffa caffa’aa 4. Lola ciisaa | | | | | | | | | | | | | | | |
|  | Ilaalcha /attitude items Himoota kanneeniif gaaffii fulduratti mallattoo ‘√” filanno deebifame jallatti mirkannessi] | | | | | | | | | | | | Itti walii hin galu =0 | | | | | Hin murteessine  =1 | | | | | Ittinwalii gala=2 | | | |  | |
| Q41.1 | Halkan hundaa miseensonni maatii hunduu saapahan siree jala rafuu qabu | | | | | | | | | | | |  | | | | |  | | | | |  | | | |  | |
| Q41.2 | Halkan hunda agoobara keessa buluun bookeen ciniinamuu ittisa | | | | | | | | | | | |  | | | | |  | | | | |  | | | |  | |
| Q41.3 | Agoobarri waan ho’uuf halkan hunda keessa buluuf hin mijatu | | | | | | | | | | | |  | | | | |  | | | | |  | | | |  | |
| Q42 | Duubartiin ulfaa fi ijoolleen waggaa shanii gadii halkan hundaa saapahan siree jala akka rafaniif dursi kennamuuf qaba. | | | | | | | | | | | |  | | | | |  | | | | |  | | | |  | |
| Q43.1 | Yoo miseensi maatii tokko qaama gubaa qabaate/tte, hatattamaan mana yaalaa naannoo isaaniitti geeffamuu qabu. | | | | | | | | | | | |  | | | | |  | | | | |  | | | |  | |
| Q43.2 | Qaama gubaan mallattoo dhibee cimaati kanaaf yaalamuu qaba | | | | | | | | | | | |  | | | | |  | | | | |  | | | |  | |
| Q43.3 | Gubaa qaama/busaaf falli barbaachisaan mana yaala qofa jira | | | | | | | | | | | |  | | | | |  | | | | |  | | | |  | |
| Q44 | Namni tokko qoricha busaa ogeeyyii fayyaan, ektenshinoota fayyaa dabalatee, ajajamee hundaa dirqama fudhachuu qaba. | | | | | | | | | | | |  | | | | |  | | | | |  | | | |  | |
| Q45 | Namni tokko qoricha busaa ogeeyyii fayyaatiin isaaf ajajame addaan kutuu ykn nama biraatiif qooduu hin qabu | | | | | | | | | | | |  | | | | |  | | | | |  | | | |  | |
| Q46 | Maatiin yeroo manni keessi biifamu warra biifan gargaaruu/deeggaruu qabu. | | | | | | | | | | | |  | | | | |  | | | | |  | | | |  | |
| Q47 | Maatiin tokko erga manni biifamee booda hanga ji’oota jahaatti manicha maariguu fi qalama dibuu hin qabnu. | | | | | | | | | | | |  | | | | |  | | | | |  | | | |  | |
| Q48 | Saapahana siree farra ilbiisotaatiin cuuphame, saamunaan miiccinee fi suuquu ykn gaddisa keessatti hafuu qabna | | | | | | | | | | | |  | | | | |  | | | | |  | | | |  | |
| Q49 | Maatiin hundi buusaa ittissuf naannoo jireenya isaanii qulqulleessuu qabu | | | | | | | | | | | |  | | | | |  | | | | |  | | | |  | |
|  | Value associated with each action : Tokkon tokko gaaff fulduratti mallattoo ‘√” filanno deebifame jallatti mirkannessi] | | | | | | | | | | | | Walii hin galu =0 | | | | | Hin-murteessine  =1 | | | | | Ittin walii gala =2 | | | |  | |
| Q50.1 | Saaphana siree jala rafuun karaa salphaa maatiin busaa irraa ittiin of eeganidha | | | | | | | | | | | |  | | | | |  | | | | |  | | | |  | |
| Q50.2 | Bookeen ciniinamuu dhabuun dhibee busaa irra nama eega | | | | | | | | | | | |  | | | | |  | | | | |  | | | |  | |
| Q50.3 | Ho’a isaa sodaatanii agoobara keessa buluu dhisuun busaaf waan saaxiluuf fayyaa namaa miidha | | | | | | | | | | | |  | | | | |  | | | | |  | | | |  | |
| Q51 | Duubartiin ulfaatiif ijoolleen waggaa shanii gadii irraa caalaa busaadhaaf saaxilamoo dha.(less resistance to fight off malaria. | | | | | | | | | | | |  | | | | |  | | | | |  | | | |  | |
| Q52.1 | Qaama gubaa ykn buusaa Yeeroodhaan dursanii yaalamuun busaan akka hin hammaanne/cimne dhoorka. | | | | | | | | | | | |  | | | | |  | | | | |  | | | |  | |
| Q52.2 | Qaama gubaaf yaalamuun rakkoo fayyaa walxaxaa ittisuu dha | | | | | | | | | | | |  | | | | |  | | | | |  | | | |  | |
| Q52.3 | Kunuunsi dhibee busaa/gubaa qaamaf karaa mana yaalaa kennamu fayyaa eeguuf filannoo dursaati | | | | | | | | | | | |  | | | | |  | | | | |  | | | |  | |
| Q53 | Guutumaan guututti busaa irraa fayyuuf qoricha busaa kan ogeeyyii fayyaatiin ajajame hundaa xumuruun barbaachisaadha | | | | | | | | | | | |  | | | | |  | | | | |  | | | |  | |
| Q54 | Qoricha busaa ajajame haalan hin fudhanne yoo ta’e dhukkubsataan hin fayyu-dhukkubnis itti deebi’u danda`a | | | | | | | | | | | |  | | | | |  | | | | |  | | | |  | |
| Q55 | Keemikaalli farra bookee busaa mana keessatti biifamu maatin mana isaanii bookee buusaa dhibee busaa daddabarsitu irraa akka eeganiif gargaara | | | | | | | | | | | |  | | | | |  | | | | |  | | | |  | |
| Q56 | Mana irra deebi`anii maariguun/halluu dibuun bu`a qabeessummaa keemikaala farra bookee busaa mana keessatti biifamu hir`isa. | | | | | | | | | | | |  | | | | |  | | | | |  | | | |  | |
| Q57 | Saaphana siree miiccun bu’aa farra-ilbiisummaa isaa qabatee akka turuuf ni gargaara. | | | | | | | | | | | |  | | | | |  | | | | |  | | | |  | |
| Q58 | Osoo warri biifan biifuudhaaf mana koo dhufanii akka biifan nan eeyyamaaf | | | | | | | | | | | |  | | | | |  | | | | |  | | | |  | |
| Q59 | Naannoo jireenya keenyaa qulqullinaan eeguun busaa ittisa | | | | | | | | | | | |  | | | | |  | | | | |  | | | |  | |
|  | Ofitti amanamummaa ilaalu/Self-efficacy items: shaakala armaan gaditti tarrefamani hammam akka ofitti amanamummaan hojiirra olchuu danda’an tokkon tokko gaaffii fulduratti mallattoo ‘√” filanno deebifame jallatti mirkannessi] | | | | | | | | | | | | Gonkuma  Lakki =0 | | | | | Hamma ta’e =1 | | | | | Gutummaguututti, eyye=2 | | | |  | |
| Q60 | Ofii koo busaa/wobaa irraa salphaatti eegu nin danda’a | | | | | | | | | | | |  | | | | |  | | | | |  | | | |  | |
| Q61 | Daa’imman kiyya salphaatti busaa irraa eeguu nin danda’a | | | | | | | | | | | |  | | | | |  | | | | |  | | | |  | |
| Q62 | Miseensi maatiikoo yoo busaan qabaman salphaatti kunuunsa kennuu nin danda’a | | | | | | | | | | | |  | | | | |  | | | | |  | | | |  | |
| Q63 | Halkan hundumaa hagoobara jala raafuuf ofitti nin amana. | | | | | | | | | | | |  | | | | |  | | | | |  | | | |  | |
| Q64 | Maatiinkoo halkan hundumaa hagoobara jala akka raafanu gochuuf ofitti nin amana. | | | | | | | | | | | |  | | | | |  | | | | |  | | | |  | |
| Q65 | Erga manni koo biifame booda ( osoo biifamee jiruuyyuu) itti fufinsaan hagoobarakootti fayyadamuu nin danda’a | | | | | | | | | | | |  | | | | |  | | | | |  | | | |  | |
| Q66 | Yeroo ho’aa/ ukkamamuun natti dhaga’amu/ hagoobara jala rafuuf ofitti amanamummaa nin qaba. | | | | | | | | | | | |  | | | | |  | | | | |  | | | |  | |
| Q67 | Jismii/qamaa gubaan mallattoo busaa/wobaa ta’eef ykn wanta biroo ta’ee isaa adda baasuu nin beeka/danda’a. | | | | | | | | | | | |  | | | | |  | | | | |  | | | |  | |
| Q68 | Miseensi maatiikoo dhibee busaa beekamaa/idilee ykn balaafamaa yoo qabatan walii maksee nin beeka | | | | | | | | | | | |  | | | | |  | | | | |  | | | |  | |
| Q69 | Miseensi maatiikoo busaa qabu jedhee yeroon yaadu, saa’atii 24 kessatti kilinikatti akkan sakkatasisuu nin ragaasisa | | | | | | | | | | | | 0 | | | | | 1 | | | | | 2 | | | |  | |
|  |  |  |  |  |  |  |  |  |  |  |  |  |  | | | | |  | | | | |  | | | |  | |
| Q70 | Bakki filatamaan jismi/qaama gubaa/busaa maatii keenyaa itti yaaluuf barbaadnu dhabbata fayyaati. | | | | | | | | | | | |  | | | | |  | | | | |  | | | |  | |
| Q70 | Miseensa maatii keessaa yeroo namni busaan qabamu, saa’atii 24 keessatti yaala barbaachisaa argachuuf nin mirkaneessa | | | | | | | | | | | |  | | | | |  | | | | |  | | | |  | |
| Q71 | Yeroo miseensi matii baayyee dhukkubsate, qabeenya saa’atii 24 kessatti gara kiliniikaa waliin deemuuf barbaachisu argachuu nin danda’a. | | | | | | | | | | | |  | | | | |  | | | | |  | | | |  | |
| Q72 | Maatiinkoo qoricha isaaniif ajajame hundumasaa akka fudhatanuu mirkaneessuuf ofitti amanamummaa ni qaba | | | | | | | | | | | |  | | | | |  | | | | |  | | | |  | |
| Q73 | Yeroo qamni biifuu dhufe, meeshaalee mana keessaa jiran gadi guuruun haalaa mijeesuu nin danda’a | | | | | | | | | | | |  | | | | |  | | | | |  | | | |  | |
| Q74 | Erga manni biifamee booda, ji’oota 6’f manicha maraguu ykn haaluu dibuu irraa of-qusadhe turuu nin danda’a | | | | | | | | | | | |  | | | | |  | | | | |  | | | |  | |
| Q75 | Haalan osoo hin daangeffamin daa’ima umrii <5 halkaan hundumaa hagoobara jala raffisuuf ofitti nin amana. | | | | | | | | | | | |  | | | | |  | | | | |  | | | |  | |
| Q76 | Haalan osoo hin daangeffamin dubartii ulfaa halkaan hundumaa hagoobara jala raffisuuf ofitti nin amana. | | | | | | | | | | | |  | | | | |  | | | | |  | | | |  | |
| Q77 | Halkan hundumaa hagoobara jala rafuun carra busaan qabamuukoo daran hir’isuu nin danda’a. | | | | | | | | | | | |  | | | | |  | | | | |  | | | |  | |
| Q78 | Halkan hundumaa agoobara jala raffisuun carraa daa’ima (<5 gadii) busaan qabamuu daran hir’isuu nin danda’a. | | | | | | | | | | | |  | | | | |  | | | | |  | | | |  | |
| Q79 | Busaa irraa maatiikoo eeguuf, naannoo jireenyakoo dhaabataan (yoo xiqqate torbeetti) qulqulleessuuf ofitti nin amana. | | | | | | | | | | | |  | | | | |  | | | | |  | | | |  | |
|  | Percieved suscetibility from risk of malaria: miidhaa dhukkubaa busaa irraan dhufa jettee yaadutti ammam saaxilamoo akka taate tokkon tokko gaaffii fulduratti mallattoo ‘√” filanno deebifame jallatti mirkannessi | | | | | | | | | | | | Itti walii hin galu =0 | | | | | Hin murteessine  =1 | | | | | Itti walii gala =2 | | | |  | |
| Q80 | Waa’ee busaa hin dhiphadhu, sabaabni isaas waan salphaatti yaalamuu dandaa’uuf. | | | | | | | | | | | |  | | | | |  | | | | |  | | | |  | |
| Q81 | Busaan hawaasa keenya keessaa badee jira | | | | | | | | | | | |  | | | | |  | | | | |  | | | |  | |
| Q82 | Busaan maatiikoof ammaan booda rakkoo miti. | | | | | | | | | | | |  | | | | |  | | | | |  | | | |  | |
| Q83 | Carraan ani busaan qabamuuf qabu guddaa akka ta’e natti dhagaa’ama.* | | | | | | | | | | | |  | | | | |  | | | | |  | | | |  | |
| Q84 | Carraan maatiin koo busaan qabamuuf qabanu guddaa akka ta’e natti dhaga’ama.* | | | | | | | | | | | |  | | | | |  | | | | |  | | | |  | |
| Q85 | Tibba waqitii roobaa keessa, maatiikoo kessaa namni tokko busaan qabamaa laata jedhee guyyuma guyyaanin yaada* | | | | | | | | | | | |  | | | | |  | | | | |  | | | |  | |
| Q86 | Namootni kan busaan qabamanu yeroo bookeen busaa heddummatu qofa dha. | | | | | | | | | | | |  | | | | |  | | | | |  | | | |  | |
| Q87 | Daa’imoonni koo fayyummaa gaarii waan qabaniif dhibee busaa dandamachuu ni danda’u. | | | | | | | | | | | |  | | | | |  | | | | |  | | | |  | |
| Q88 | Namoonni hawaasa kanaa waqtii roobaa qofa busaan qabamu | | | | | | | | | | | |  | | | | |  | | | | |  | | | |  | |
| Q89 | Yeroo dhiyootti namni ani beekuu kan busaan garmalee dhukkubsate hin yaadadhu | | | | | | | | | | | |  | | | | |  | | | | |  | | | |  | |
| Q90 | Waguma waggaan jechuun ni danda’ama, namni hawaasa kanaa dhibee busaa cimaa/balafamaan dhukkubsata* | | | | | | | | | | | |  | | | | |  | | | | |  | | | |  | |
| Q91 | Dubartiin ulfaa minseesa hawaasichaa kan biro irraa rakkoon addaa karaa busaa ishee mudatu hin jiru. | | | | | | | | | | | |  | | | | |  | | | | |  | | | |  | |
|  | Percieved severity of risk of malaria : [Tokkon tokko gaaffii fulduratti mallattoo ‘√” filanno deebifame jallatti mirkannessi] | | | | | | | | | | | | Itti walii hin galu =0 | | | | | Hin murteessine  =1 | | | | | Itti walii gala =2 | | | |  | |
| Q92 | Dhibee busaatiin qabamuun du’aaf nama geessisuu ni danda’a | | | | | | | | | | | |  | | | | |  | | | | |  | | | |  | |
| Q93 | Yeroo daa’imni koo qaama gubaa qabaatu, yeroo hundumaa busaa ta’uu dand’a jedheen dhipadha | | | | | | | | | | | |  | | | | |  | | | | |  | | | |  | |
| Q94 | Namni ani beekuu tokko yeroo busaan qabamu, yeroo baay’ee guyyota yaartuu kessatti itti fura jedheen tilmaama* | | | | | | | | | | | |  | | | | |  | | | | |  | | | |  | |
| Q95 | Yeroo daa’imni koo qaama gubaa qabaatu, osoo gara ogeessa fayyaatti in imalin duraa guyyota muraasaaf nan obsa* | | | | | | | | | | | |  | | | | |  | | | | |  | | | |  | |
| Q96 | Yeroo dubartiin ulfaa busaan qabamtu, ishee fi ulfi garaa kessa jiru walxaxinsaa busaan heddu miidhamuu ni danda’u. | | | | | | | | | | | |  | | | | |  | | | | |  | | | |  | |
|  | Ergaalee busaaf saaxilamuu (Exposure and recall of malaria messages) | | | | | | | | | | | | | | | | | | | | | | | | | |  | |
| Q97 | Ji`ooottan 6`n darban keessatti waa`ee busaa fi ittisa isaa irraatti odeeffannoo fudhattee beektaa? | | | | | | | | | | | 1. Eeyyee 2. Lakki→gara Q100tti tari | | | | | | | | | | | | | | |  | |
| Q98 | Eessaa dhageesse?  Filannoolee hin dubbisin  Altokko irra deebi’ii ‘’wanti biroo kan hafe jiraa?’’ Jedhii gaafadhu  Deebii sitti himamu hunda itti mari | | | | | | | | | | | 1. Barattoota irraa- maatii keessaa 2. Barattoota irraa- maatiin ala 3. Gaggeessitoota amantaa Magiidarra 4. Gaggeesstoota amantaa waldaa kiristaanaa irraa 5. Gaggeesstoota amantaa iddoo hawaasni walitti qabaman irraa 6. Barsiisota irraa 7. Hojjettoota ekteenshinii fayyaa irraa 8. Hojjettoota/ogeessota fayyaa irrraa 9. Raayyaa misooma fayyaa irraa 10. Ollaa /hiriyaa irraa 11. Poostara/biilboordii irraa 12. Televizhiinii irraa 13. Raadiyoo 14. Kanneen biroo 15. Karaa walgayii walgayii gandaatiin 16. Kanneen biroo ____________ | | | | | | | | | | | | | | |  | |
| Q99 | Ergaa busaa isa kam dhageesse?  Altokko irra deebi’ii ‘’wanti biroo kan hafe jiraa?’’ Jedhii gaafadhu  Deebii sitti himamu hunda itti mari  DEEBII HEDDUUN NI DANDA`AMA | | | | | | | | | | | 1. Halkan hundumaa saapahana siree jala rafuu 2. Saaphana sirree fayyadamuu irratti dursa haadholii ulfaa fi daa`imman waggaa shanii gadiif kennuu 3. Qoricha farra busaa haalan fayyadamuu 4. Yeroo keemikaala farra bookee busaa mana keessatti biifnu ofeeggannoo gochuu 5. Akkaataa itti saaphanni siree miiccamu 6. Qaama gubaaf hatattamaan kunuunsa barbaaduu 7. Naannoo jireenyaa qulqulleessuun busaa ittisuu 8. Kanneen biroo____________ 9. Hin yaadadhu | | | | | | | | | | | | | | |  | |
| Q100 | Daa`ima (miseesa maatii kan ta’e) barnoota baratu qabda? | | | | | | | | | | | 1. Eeyyee 2. Lakki →Gaaffii 104 deemi | | | | | | | | | | | | | | |  | |
| Q101 | Eeyyee yoo ta’e, baayina daa’ima barataa umurii, saala fi kutaa isaan baratanu waaliin caqasi  [xiqqadha gara guddatti tarasi] | | | | | | | | | | | \|  \| D-1 \| D-2 \| D-3 \| D-4 \| \| --- \| --- \| --- \| --- \| --- \| \| Umuri \|  \|  \|  \|  \| \| Saala \|  \|  \|  \|  \| \| Kutaa \|  \|  \|  \|  \| | | | | | | | | | | | | | | |  | |
| Q102 | Daa`imni kee waa`ee busaa waan tokkoyyuu sitti himee beeka? | | | | | | | | | | | 1. Eeyyee 2. Lakki | | | | | | | | | | | | | | |  | |
| Q103 | Ergaa waa`ee busaa maal sitti hime/te?  Altokko irra deebi’ii ‘’wanti biroo kan hafe jiraa?’’ Jedhii gaafadhu  Deebii sitti himamu hunda itti mari  DEEBII HEDDUUN NI DANDA`AMA | | | | | | | | | | | 1. Halkan hundumaa saapahana siree jala rafuu 2. Saaphana sirree fayyadamuu irratti dursa haadholii ulfaa fi daa`imman waggaa shanii gadiif kennuu 3. Qoricha farra busaa haalan fayyadamuu 4. Yeroo keemikaala farra bookee busaa mana keessatti biifnu ofeeggannoo gochuu 5. Akkaataa itti saaphanni siree miiccamu fi kuununfamu 6. Qaama gubaaf hatattamaan kunuunsa barbaaduu 7. Naannoo jireenyaa qulqulleessuun busaa ittisuu 8. Kanneen biroo____________ 9. Hin yaadadhu | | | | | | | | | | | | | | |  | |

| Raga sassaabaa/duu | | To`ataa/ttu/suparvaayizaraa | Bu`aa af-gaaffii |
| --- | --- | --- | --- |
| Maqaa: |  | maqaa: | 1. Xumurameera 2. Walakkaan isaa guutameera 3. Ni didan/didame 4. Kan biraa[ibsi] |
| Guyyaa : |  | Guyyaa: _____________ |  |
| Mallattoo: |  | Mallattoo: |  |
