# Supplementary material for: School-based social and behavior change communication (SBCC) advances community exposure to malaria messages, acceptance, and preventive practices in Ethiopia: A pre-posttest study
Source: PLoS One. 2020 Jun 25;15(6):e0235189. doi: 10.1371/journal.pone.0235189 (PMC7316301; doi:10.1371/journal.pone.0235189)
Supplement: S5 File — (DOCX) [file pone.0235189.s005.docx]

**Household repeated survey questionnaires**

**Malaria Knowledge, Attitude, perceptions, behaviors, and exposure to messages**

| Read informed consent for household questionnaire | | | | | | | | | | | | | | | | | | | | | | | | | |  | | | |
| --- | --- | --- | --- | --- | --- | --- | --- | --- | --- | --- | --- | --- | --- | --- | --- | --- | --- | --- | --- | --- | --- | --- | --- | --- | --- | --- | --- | --- | --- |
| Respondent agrees to be interviewed 1  🡫interview | | | | | Respondent does not agree to be interviewed……2 ──end | | | | | | | | | | | | | | | | | | | | |  | | | |
|  | Identification | | | | | | | | | | | | | | | | | | | | | | | | | | | | |
|  | Survey status: 1) Baseline 2) Follow-up (end line) | | | | | | | | | | | | | | | | | | | | | | | | | | | | |
|  | District name | | | ______________________ | | | | | | | | | | | | | | | | | | | | |  | | | | |
|  | Altitude at the center of the kebele | | |  | | | | | | | | | | | | | | | | | | | | |  | | | | |
|  | Kebele name | | | ______________________ | | | | | | | | | | | | | | | | | | | | |  | | | | |
|  | Name of head of household | | | ______________________ | | | | | | | | | | | | | | | | | | | | |  | | | | |
|  | Zone | | | ______________________ | | | | | | | | | | | | | | | | | | | | |  | | | | |
|  | Setting | | | 1. Urban 2. Rural | | | | | | | | | | | | | | | | | | | | |  | | | | |
|  | Interview Identification | | | DD/KK/HH/IN | | | | | | | | | | | | | | | | | | | | |  | | | | |
|  | Respondents background | | | | | | | | | | | | | | | | | | | | | | | | | | | | |
| Q01 | What is your age? | | | _______[completed years] | | | | | | | | | | | | | | | | | | | | |  | | | | |
| Q02 | Sex | | | 1. Male 2. Female | | | | | | | | | | | | | | | | | | | | |  | | | | |
| Q03 | Is the head of household? | | | 1. Male 2. Female | | | | | | | | | | | | | | | | | | | | |  | | | | |
| Q04 | Marital status | | | 1. Married-living together 2. Married-living separately 3. Divorced 4. Widowed 5. other(specify)_________ | | | | | | | | | | | | | | | | | | | | |  | | | | |
| Q05 | How many wife does your husband have? | | | ______________________ | | | | | | | | | | | | | | | | | | | | |  | | | | |
| Q06 | Education status | | | 1. Can’ read and write 2. Read and write but no formal education 3. _____[Highest grade completed if attended school] | | | | | | | | | | | | | | | | | | | | |  | | | | |
| Q07 | Spouse educational status, if alive | | | 1. Can’ read and write 2. Read and write but no formal education 3. _____[Highest grade completed if attended school] 4. Not applicable | | | | | | | | | | | | | | | | | | | | |  | | | | |
| Q08 | Religion | | | 1. Muslim 2. Orthodox 3. Protestant 4. Other [specify]________ | | | | | | | | | | | | | | | | | | | | |  | | | | |
| Q09 | Ethnicity | | | 1. Oromo 2. Ahmara 3. Yam 4. Dawuro 5. Other [specify]__________ | | | | | | | | | | | | | | | | | | | | |  | | | | |
| Q10 | Respondent’s occupation | | | 1. Farmer 2. Merchant 3. Government employ 4. Private business 5. Other [specify]_________ | | | | | | | | | | | | | | | | | | | | |  | | | | |
| Q11 | Family Size [permanent member, who lived for >=6 months] | | | _____________________________ | | | | | | | | | | | | | | | | | | | | |  | | | | |
|  | Knowledge related items | | | | | | | | | | | | | | | | | | | | | | | | | | | | |
| Q12 | Have you ever heard of or know an illness called malaria/woba/busa? | | | 1. Yes 2. No | | | | | | | | | | | | | | | | | | | | |  | | | | |
| Q13 | Can you tell me the main symptoms of malaria?  Don’t read options  MULTIPLE RESPONSES  PROBE ONCE (Anything else?) | | | 1. Fever 2. Feeling cold 3. Headache 4. Nausea and Vomiting 5. Diarrhea 6. Dizziness 7. Loss of appetite/refuse to eat/drink 8. Body ache or joint pain 9. Pale eyes 10. Body weakness 11. Other (Specify)_________ 12. Don’t know | | | | | | | | | | | | | | | | | | | | |  | | | | |
| Q14 | In your opinion, what causes malaria?  Don’t’ read option  MULTIPLE RESPONSES  PROBE ONCE (Anything else?) | | | 1. Mosquito bites 2. Eating maize 3. Eating sugarcane 4. hunger (empty stomach) 5. Eating/drinking other dirty food/water 6. Getting soaked with rain 7. Cold or changing weather 8. Lack of hygiene 9. Other (Specify)________ 10. don’t know | | | | | | | | | | | | | | | | | | | | |  | | | | |
| Q15 | How can a person protect themselves against malaria?  Don’t read options  MULTIPLE RESPONSES  PROBE ONCE (Anything else?) | | | 1. Sleep under a mosquito net 2. Using repellants 3. Spray house with insecticide 4. Keep house surroundings clean 5. Fill in puddles (stagnant water) 6. Don’t get soaked with rain 7. Other (Specify)________ 8. Don’t know | | | | | | | | | | | | | | | | | | | | |  | | | | |
| Q16 | In your opinion, who is most likely to get a serious case of “malaria” among families?    [read options to choose only two target groups] | | | 1. Adult man 2. Adult women 3. Pregnant women 4. A child of six years old 5. A child of 3 years old 6. don’t know | | | | | | | | | | | | | | | | | | | | |  | | | | |
|  | Ownership and access to Insecticide treated mosquito nets | | | | | | | | | | | | | | | | | | | | | |  | | | | | | |
| Q17 | Does your household have mosquito nets that can be used while sleeping? | | | 1. Yes 2. No | | | | | | | | | | | | | | | | | | | If 2, skip to Q23 | | | | | | |
| Q18 | How many mosquito nets does your household have? | | | ______[number of nets] | | | | | | | | | | | | | | | | | | |  | | | | | | |
|  |  | | | N1 | | | | | N2 | | | N3 | | | N4 | | | | N5 | | | | N6 | | | | | N7 | |
| Q19 | Ask respondent to show you the net(s) in the household and fill the following table for each net. If more than 7 nets, draw another column.   1. Observed 2. Not observed | | |  | | | | |  | | |  | | |  | | | |  | | | |  | | | | |  | |
| Q20 | Has the net hole or torn or damaged?   1. Yes 2. No | | |  | | | | |  | | |  | | |  | | | |  | | | |  | | | | |  | |
| Q21 | In what condition is the net? [observe]   - - - 1. Over the bed/mat       2. Kept folded   Used for other purpose | | |  | | | | |  | | |  | | |  | | | |  | | | |  | | | | |  | |
| Q22 | How long ago did your household obtain the mosquito net? [In months | | |  | | | | |  | | |  | | | |  | | |  | | | |  | | | | |  | |
|  | ITN utilization behaviors | | | | | | | | | | | | | | | | | |  | | | | | | | | | | |
| Q23A | I would like to have some information about the people who live in your household. Could you give me first age, sex of members of the household? And then I will ask you also who and how they use mosquito net. Please start with the youngest including babies. (RECORD, even for households which does not have nets) [Interviewer: Record from the youngest through the oldest] | | | | | | | | | | | | | | | | | | | | | | | | | | | | |
|  | ANSWERS IN THE COLUMN FOR THE FOLLOWING QUESTIONS) | | | | | | | | | | | | | | | | | | | | | | | | | | | | |
| Code | Name | | Age  (No. Of Years) | | | | Sex  M=1  F=2 | | | | | | Pregnant  Yes=1  No=0  [If women age b/n 15-49) | | | | | | | | Slept under net previous night?  Yes=1  No=0  (if the respondent was away that night he/she can ask others who were at home in previous night) | | | | | | | | |
|  |  | |  | | | |  | | | | | |  | | | | | | | |  | | | | | | | | |
|  |  | |  | | | |  | | | | | |  | | | | | | | |  | | | | | | | | |
|  |  | |  | | | |  | | | | | |  | | | | | | | |  | | | | | | | | |
|  |  | |  | | | |  | | | | | |  | | | | | | | |  | | | | | | | | |
|  |  | |  | | | |  | | | | | |  | | | | | | | |  | | | | | | | | |
|  |  | |  | | | |  | | | | | |  | | | | | | | |  | | | | | | | | |
|  |  | |  | | | |  | | | | | |  | | | | | | | |  | | | | | | | | |
|  |  | |  | | | |  | | | | | |  | | | | | | | |  | | | | | | | | |
|  |  | |  | | | |  | | | | | |  | | | | | | | |  | | | | | | | | |
|  |  | |  | | | |  | | | | | |  | | | | | | | |  | | | | | | | | |
|  |  | |  | | | |  | | | | | |  | | | | | | | |  | | | | | | | | |
|  |  | |  | | | |  | | | | | |  | | | | | | | |  | | | | | | | | |
|  |  | |  | | | |  | | | | | |  | | | | | | | |  | | | | | | | | |
| Q23B | Q23.B) In this house, how many sleeping arrangements/spaces are there (bed and separate rooms are not mandatory to count a space)?_________( put the number) | | | | | | | | | | | | | | | | | | | | | | | | | | | | |
| Q23C | Q23 C) Last night who slept over which sleeping arrangement (add extra column if more than 4  persons per sleeping spaces)   \| *Ask for sleeping arrangements* \| Space-1 \| Space-2 \| Space-3 \| Space 4- \| \| --- \| --- \| --- \| --- \| --- \| \| 1. Number of persons (##) \| ____________ \| ___________ \| ________ \| ____________ \| \| 1. Sex (##) \| M___F___ \| M____F___ \| M_____F__ \| M____F___ \| \| 1. Pregnant women \| _______ \| _________ \| ________ \| _________ \| \| 1. Children <5 age \| _______ \| _________ \| ________ \| __________ \| \| 1. Has ITN (Yes=1, No=0) \| _______ \| _________ \| ________ \| __________ \| | | | | | | | | | | | | | | | | | | | | | | | | | | | | |
| Q23D | (ITNs net care knowledge) | | | | | | | | | | | | | | | | | | | | | | | | | | | | |
|  | While washing ITN, pick all actions to refrain from the following lists | | | | | | | 1. Avoid soap with detergent effect 2. Avoid washing on hard and rocky surface 3. Avoid usingt brush while washing 4. Avoid dumping in waterfor long 5. Avoid exposure to sunlight 6. Avoid washing within 3 months 7. Other ______________ 8. I don’t know 9. ITN should not washed at all | | | | | | | | | | | | | | | | | | |  | | |
|  | When ITN is washed its insecticidal effect will reduce | | | | | | | 1. True 2. False 3. I don’t know | | | | | | | | | | | | | | | | | | |  | | |
|  | What should be done in order to care for ITN to prevent untimely damage? | | | | | | | 1. Tie as suspend every day 2. Wisely enter to get under it 3. Do not unnecessarly stretch 4. Follow up on daily basis 5. Prevent children from playing inside 6. Other ____________ 7. I don’t know | | | | | | | | | | | | | | | | | | |  | | |
|  | What are mechanisms to amend ITN when it has torn or tear to make it functional? | | | | | | | 1. Use needle and thread to stich 2. Use any material to cover any hole 3. Use small clock to stick the hole 4. Other ____________________ 5. I don’t know | | | | | | | | | | | | | | | | | | |  | | |
|  | Treatment seeking behaviors | | | | | | | | | | | | | | | | | | | | | | | | | | | | |
| Q24 | Was there any person who sick from fever in the household during the last 2 weeks? | | | | | | | | | | | | | | | | | | | 1. Yes 2. No | | | | | | If 2 skip to → Q37 | | | |
| Q25 | If yes, fill the following table. If more than four person, use another questionnaire | | | | | | | | | | | | | | | | | | | | | | | | | | | | |
|  |  | | | | | | | | | | | | | | | | P 1 | | | P 2 | | | | P 3 | | | | P4 | |
|  | Code | | | | | | | | | | | | | | | |  | | |  | | | |  | | | |  | |
|  | Age | | | | | | | | | | | | | | | |  | | |  | | | |  | | | |  | |
|  | Sex | | | | | | | | | | | | | | | |  | | |  | | | |  | | | |  | |
| Q26 | How many days ago did the fever start? | | | | | | | | | | | | | | | |  | | |  | | | |  | | | |  | |
| Q27 | Is (NAME) still sick with a fever?   1. yes 2. No | | | | | | | | | | | | | | | |  | | |  | | | |  | | | |  | |
| Q28 | Did the person seek advice or treatment for the fever from any source?   1. Yes 2. No | | | | | | | | | | | | | | | |  | | |  | | | |  | | | |  | |
| Q29 | Where did you seek advice or treatment?   1. Health Post 2. Health center 3. Hospital 4. Pharmacy 5. Private clinic 6. Other[specify]_____   Anywhere else?  RECORD ALL SOURCES MENTIONED. | | | | | | | | | | | | | | | |  | | |  | | | |  | | | |  | |
| Q30 | How many days after the fever began did [NAME] first seek advice or treatment?  In Days | | | | | | | | | | | | | | | |  | | |  | | | |  | | | |  | |
| Q31 | At any time during the illness, did (NAME) take any drugs for the fever?   1. Yes 2. No | | | | | | | | | | | | | | | |  | | |  | | | |  | | | |  | |
| Q32 | What drugs did (NAME) take?  Any other drugs? SHOW THE DRUGS & ASK FOR WHICH DRUG THEY TAKE.   1. Coartem 2. Chloroquine 3. quinine 4. Percetamol 5. Ibuprofen 6. other_____ 7. Don’t know   RECORD ALL MENTIONED FOR EACH PERSON | | | | | | | | | | | | | | | |  | | |  | | | |  | | | |  | |
| Q33 | Did you have the anti-malarial drug at home or did you get it from somewhere else? IF SOMEWHERE ELSE, PROBE FOR SOURCE.  IF MORE THAN ONE SOURCE MENTIONED, ASK:  Where did you get the drugs? (ask for anti-malarial drug alone)   1. at home 2. Health post 3. Health center 4. private clinic 5. shop 6. other_____ 7. don’t know | | | | | | | | | | | | | | | |  | | |  | | | |  | | | |  | |
| Q34 | Does currently [call name} taking the drug?   1. Yes-taking 2. yes-completed 3. Discontinued 4. Not used any drug | | | | | | | | | | | | | | | |  | | |  | | | |  | | | |  | |
| Q35 | Did you share some of your drugs for someone else?   1. Yes 2. No 3. Not received any drug | | | | | | | | | | | | | | | |  | | |  | | | |  | | | |  | |
| Q36 | Was blood taken and checked during fever, if seen at health facility?   1. Yes 2. No 3. Not visited any health facility | | | | | | | | | | | | | | | |  | | |  | | | |  | | | |  | |
|  | Indoor Residual Spray practice | | | | | | | | | | | | | | | | | | | | | | | | | | |  | |
| Q37 | At any time in the past 12 months, has anyone sprayed the interior walls of your dwelling against mosquitoes? | | | | | | | | | | 1. Yes 2. No →Q41 3. Don’t know→Q41 | | | | | | | | | | | | | | | | |  | |
| Q38 | How many months ago was the house sprayed? | | | | | | | | | | Months ago______ | | | | | | | | | | | | | | | | |  | |
| Q39 | At any time in the past 12 months, have the walls in your dwelling been plastered or painted? | | | | | | | | | | 1. Yes 2. No 3. Don’t know | | | | | | | | | | | | | | | | |  | |
| Q40 | How many months ago were the walls plastered or painted? | | | | | | | | | | Months ago______________ | | | | | | | | | | | | | | | | |  | |
|  | Attitude items  Interviewer: Now I ask you to tell me whether you agree or disagree to the following statements [Read the responses & check‘√” infront of each question under the responded option] | | | | | | | | | | Disagree=0 | | | Undecided  =1 | | | | | | | | Agree =2 | | | | | |  | |
| Q41 | All family members should sleep under ITNs, every night. | | | | | | | | | |  | | |  | | | | | | | |  | | | | | |  | |
| Q42 | Pregnant women and children under five (U5) should be given priority to sleep under ITNs, every night. | | | | | | | | | |  | | |  | | | | | | | |  | | | | | |  | |
| Q43 | Whenever a family member has a fever, they must be taken to the nearest health facility, immediately. | | | | | | | | | |  | | |  | | | | | | | |  | | | | | |  | |
| Q44 | One must take full dose of the anti-malaria drugs prescribed to him by health personnel, including HEWs. | | | | | | | | | |  | | |  | | | | | | | |  | | | | | |  | |
| Q45 | One should not interrupt or share his/her anti-malaria drugs prescribed to him/her by health personnel. | | | | | | | | | |  | | |  | | | | | | | |  | | | | | |  | |
| Q46 | A family should cooperate with sprayers during indoor residual spraying (IRS) period. | | | | | | | | | |  | | |  | | | | | | | |  | | | | | |  | |
| Q47 | A family should not re-plaster their home for six months after your house has been sprayed. | | | | | | | | | |  | | |  | | | | | | | |  | | | | | |  | |
| Q48 | One could wash his/her LLIN with „regular‟ soap and hang or lay to dry in the shade. | | | | | | | | | |  | | |  | | | | | | | |  | | | | | |  | |
| Q49 | All families should keep clean their dwelling area to prevent malaria | | | | | | | | | |  | | |  | | | | | | | |  | | | | | |  | |
|  | Value associated with each action [check ‘√” infront of each question under the responded option) | | | | | | | | | | Disagree=0 | | | Undecided  =1 | | | | | | | | Agree =2 | | | | | |  | |
| Q50 | Sleeping under ITNs is the easiest way to protect your family against malaria. | | | | | | | | | |  | | |  | | | | | | | |  | | | | | |  | |
| Q51 | Pregnant women and children under five are the most vulnerable to malaria. They have less resistance to fight off malaria. | | | | | | | | | |  | | |  | | | | | | | |  | | | | | |  | |
| Q52 | Early treatment will prevent malaria from becoming more dangerous. | | | | | | | | | |  | | |  | | | | | | | |  | | | | | |  | |
| Q53 | It is important to finish all your anti-malaria drugs as prescribed by health personnel, to be completely cured from malaria. | | | | | | | | | |  | | |  | | | | | | | |  | | | | | |  | |
| Q54 | If the prescribed anti-malaria drug is not taken appropriately, patients will not be cured and the disease could relapse | | | | | | | | | |  | | |  | | | | | | | |  | | | | | |  | |
| Q55 | IRS helps families keep their house free from malaria transmitting vector mosquitoes. | | | | | | | | | |  | | |  | | | | | | | |  | | | | | |  | |
| Q56 | Re-plastering reduces the effectiveness of the IRS. | | | | | | | | | |  | | |  | | | | | | | |  | | | | | |  | |
| Q57 | Washing your LLIN will help to maintain the effectiveness of the insecticide. | | | | | | | | | |  | | |  | | | | | | | |  | | | | | |  | |
| Q58 | I would allow if sprayer comes to my home to spray my home | | | | | | | | | |  | | |  | | | | | | | |  | | | | | |  | |
| Q59 | Keeping clean our dwelling area, prevents malaria | | | | | | | | | |  | | |  | | | | | | | |  | | | | | |  | |
|  | Self-efficacy items:  Now, I ask you to tell me how confident you are that you could/couldn’t definitely/probably do the following serious of actions. [Read the responses, & check ‘√” infront of each question under the responded option] | | | | | | | | | | Definitely, Not=0 | | | Probably=1 | | | | | | | | Definitely Yes=2 | | | | | | |  |
| Q60 | I can easily protect myself from getting malaria | | | | | | | | | |  | | |  | | | | | | | |  | | | | | | |  |
| Q61 | I can easily protect my children from getting malaria | | | | | | | | | |  | | |  | | | | | | | |  | | | | | | |  |
| Q62 | I can easily take care of my family members if they contract malaria | | | | | | | | | |  | | |  | | | | | | | |  | | | | | | |  |
| Q63 | I am confident to sleep under a bed net for the entire night | | | | | | | | | |  | | |  | | | | | | | |  | | | | | | |  |
| Q64 | I am confident to make my family sleep under a bed net every night | | | | | | | | | |  | | |  | | | | | | | |  | | | | | | |  |
| Q65 | I can continue to use my bed net after the house has been sprayed ( even when sprayed) | | | | | | | | | |  | | |  | | | | | | | |  | | | | | | |  |
| Q66 | I can discipline to sleep well under a bed net when the weather is warm/feel suffocating | | | | | | | | | |  | | |  | | | | | | | |  | | | | | | |  |
| Q67 | I Know to detect if a fever is a sign of malaria or something else | | | | | | | | | |  | | |  | | | | | | | |  | | | | | | |  |
| Q68 | I Know if any family member has a typical or serious case of malaria | | | | | | | | | |  | | |  | | | | | | | |  | | | | | | |  |
| Q69 | I am sure that I will request a diagnostic test at the clinic within 24 hrs when I think members of my family might have malaria | | | | | | | | | |  | | |  | | | | | | | |  | | | | | | |  |
| Q70 | The best place to seek treatment for a fever/malaria for family is in health facility | | | | | | | | | |  | | |  | | | | | | | |  | | | | | | |  |
| Q70 | I am sure to get the appropriate treatment within 24 hrs for any member of my household when s/he has malaria | | | | | | | | | |  | | |  | | | | | | | |  | | | | | | |  |
| Q71 | I can find resources to travel with my family member to the clinic within 24 hrs when he/she is very sick | | | | | | | | | |  | | |  | | | | | | | |  | | | | | | |  |
| Q72 | I am confident to make sure member s of my family takes the full dose of medicine that s/he is prescribed | | | | | | | | | |  | | |  | | | | | | | |  | | | | | | |  |
| Q73 | I can manage moving all my furniture out of my house to prepare the house for spraying when the personnel comes | | | | | | | | | |  | | |  | | | | | | | |  | | | | | | |  |
| Q74 | I can wait not to replaster or repaint the walls of my house after the spraying, for 6 months | | | | | | | | | |  | | |  | | | | | | | |  | | | | | | |  |
| Q75 | I am confident to make every <5 children of mine to sleep under a bed net every night regardless nuisances. | | | | | | | | | |  | | |  | | | | | | | |  | | | | | | |  |
| Q76 | I am confident pregnant woman in this household will sleep under a bed net every night regardless of nuisances. | | | | | | | | | |  | | |  | | | | | | | |  | | | | | | |  |
| Q77 | I can significantly reduce my chances of getting malaria by sleeping under a bed net every night | | | | | | | | | |  | | |  | | | | | | | |  | | | | | | |  |
| Q78 | I can significantly reduce that my <5 child has to get malaria by sleeping under a bed net every night | | | | | | | | | |  | | |  | | | | | | | |  | | | | | | |  |
| Q79 | I can disciple to clean up my dwelling areas regularly (at least weekly) to protect my family from malaria | | | | | | | | | |  | | |  | | | | | | | |  | | | | | | |  |
|  | Percieved suscetibility from risk of malaria  Now, I ask your beliefs about the likelihood of experiencing negative or harmful consequences from malaria. [Read the responses, & check ‘√” infront of each question under the responded option] | | | | | | | | | | Disagree=0 | | | Undecided  =1 | | | | | | | | Agree  =2 | | | | | | |  |
| Q80 | I don’t worry about malaria because it can be easily treated | | | | | | | | | |  | | |  | | | | | | | |  | | | | | | |  |
| Q81 | Malaria has disappeared in our community | | | | | | | | | |  | | |  | | | | | | | |  | | | | | | |  |
| Q82 | Malaria is no more risk to my family | | | | | | | | | |  | | |  | | | | | | | |  | | | | | | |  |
| Q83 | I feel that the chances are high that I can get malaria | | | | | | | | | |  | | |  | | | | | | | |  | | | | | | |  |
| Q84 | I feel that the chances are high that my family can get malaria | | | | | | | | | |  | | |  | | | | | | | |  | | | | | | |  |
| Q85 | During the rainy season, I worry almost every day that someone in my family may get malaria* | | | | | | | | | |  | | |  | | | | | | | |  | | | | | | |  |
| Q86 | People only get malaria when there are lots of mosquitoes | | | | | | | | | |  | | |  | | | | | | | |  | | | | | | |  |
| Q87 | My children are so healthy that they would be able to recover from a case of malaria | | | | | | | | | |  | | |  | | | | | | | |  | | | | | | |  |
| Q88 | People in this community only get malaria during rainy season | | | | | | | | | |  | | |  | | | | | | | |  | | | | | | |  |
| Q89 | I cannot remember the last time someone I know became dangerously sick with malaria | | | | | | | | | |  | | |  | | | | | | | |  | | | | | | |  |
| Q90 | Nearly every year, someone in this community gets a serious case of malaria* | | | | | | | | | |  | | |  | | | | | | | |  | | | | | | |  |
| Q91 | A pregnant women is at no more risk of malaria than any other member of the community | | | | | | | | | |  | | |  | | | | | | | |  | | | | | | |  |
|  | Percieved severity of risk of malaria  [check ‘√” infront of each question under the responded option) | | | | | | | | | | Disagree  =0 | | | Undecided  =1 | | | | | | | | Agree  =2 | | | | | | |  |
| Q92 | Infections with malaria can potentially lead to death | | | | | | | | | |  | | |  | | | | | | | |  | | | | | | |  |
| Q93 | When my child has a fever, I almost always worry that it might be malaria | | | | | | | | | |  | | |  | | | | | | | |  | | | | | | |  |
| Q94 | When someone I know gets malaria, I usually expect them to completely recover in a few days* | | | | | | | | | |  | | |  | | | | | | | |  | | | | | | |  |
| Q95 | When my child has a fever, I usually wait a couple of days before going to a health provider* | | | | | | | | | |  | | |  | | | | | | | |  | | | | | | |  |
| Q96 | When pregnant woman gets malaria, she and her fetus are seriously affected from complications | | | | | | | | | |  | | |  | | | | | | | |  | | | | | | |  |
|  | Exposure and recall of malaria messages | | | | | | | | | | | | | | | | | | | | | | | | | | | |  |
| Q97 | During the past 6 months, have you ever received information about malaria and its prevention measures? | | | | | | | | | 1. Yes 2. No→100 | | | | | | | | | | | | | | | | | | |  |
| Q98 | Where did you hear?  Don’t read options  Probe once  Circle all response the person provide | | | | | | | | | 1. School students-within family 2. School students-outside family 3. Religious leaders at mosque 4. Religious leaders at church 5. Religious leaders at public/social gathering 6. School teachers 7. HEWs 8. Health workers 9. Health development army 10. Neighbor/friends 11. Poster/billboards 12. TV 13. Radio 14. Others 15. Through kebele meeting 16. Others_________________ | | | | | | | | | | | | | | | | | | |  |
| Q99 | Which malaria message did you hear?  Probe once  Circle all responses given  Multiple answers are possible | | | | | | | | | 1. Sleeping under misquote every night 2. Giving priority for pregnant women and children under five on LLIN use 3. Appropriate use of anti-malarial drugs 4. Care during IRS 5. How to wash ITN 6. Seeking care for fever immediately 7. Cleaning living area to prevent malaria 8. Others ____________ 9. Don’t remember | | | | | | | | | | | | | | | | | | |  |
| Q100 | Do you have any child (member of a household) who is attending school? | | | | | | | | | 1. Yes 2. No →End | | | | | | | | | | | | | | | | | | |  |
| Q101 | If yes, state the number of school children with their age, sex & grade  [Record from younger to older child] | | | | | | | | | \|  \| Ch-1 \| Ch-2 \| Ch-3 \| Ch-4 \| \| --- \| --- \| --- \| --- \| --- \| \| Age \|  \|  \|  \|  \| \| Sex \|  \|  \|  \|  \| \| grade \|  \|  \|  \|  \| | | | | | | | | | | | | | | | | | | |  |
| Q102 | Did any of your child tell anything about malaria? | | | | | | | | | 1. Yes 2. No | | | | | | | | | | | | | | | | | | |  |
| Q103 | Which malaria message did you hear?  Probe once  Circle all responses given  Multiple answers are possible | | | | | | | | | 1. Sleeping under misquote every night 2. Giving priority for pregnant women and children under five on LLIN use 3. Appropriate use of anti-malarial drugs 4. Care during IRS 5. How to wash ITN 6. Seeking care for fever immediately 7. Cleaning living area to prevent malaria 8. Others ____________ 9. Don’t remember | | | | | | | | | | | | | | | | | | | End |
| Q |  | | | | | | | | |  | | | | | | | | | | | | | | | | | | |  |
| Data collector | | | | | | Supervisor | | | | | | | | | | | | Result of interview | | | | | | | | | | | |
| Name: | |  | | | | Name: | | | | | | | | | | | | 1. Completed 2. Partially completed 3. Refused 4. Other [specify] | | | | | | | | | | | |
| Date: | |  |  |  |  | Date: _____________ | | | | | | | | | | | |  |  |  |  |  |  |  |  |  |  |  |  |
| Signature: | |  | | | | Signature: | | | | | | | | | | | |  |  |  |  |  |  |  |  |  |  |  |  |
